# Membrane Biofouling: Current Understanding and Future Perspectives- An Overview

**DOI:** 10.1007/s00284-026-04882-6

**Published:** 2026-04-14

**Authors:** N. Phillip, A. A. Muleja, M. M. Motsa, B. B. Mamba, T. T. I. Nkambule, C. S. Tshangana

**Affiliations:** Institute for Nanotechnology and Water Sustainability, College of Science, Engineering and Technology, University of South Africa, Johannesburg, 1709 South Africa

## Abstract

Membrane technology is a compelling alternative for addressing some water quality issues on a global scale. Despite the demonstrated success of membrane technology, biofouling remains a persistent problem in membrane filtration processes. The impact of biofouling compromises the application and efficiency of the filtration processes by shortening membrane lifespan, reducing permeate output, increasing the energy requirements, and increasing the transmembrane pressure. All these factors combined impact treatment plant operations. Biofouling is reported to contribute ~ 45% of all membrane fouling and between 20 and 30% of operating costs. This review critically examines the factors driving membrane biofouling and evaluates conventional mitigation strategies, highlighting their limited long-term effectiveness, high operational costs, and environmental drawbacks. Various technologies discussed in this paper have been developed to mitigate biofouling; however, due to the complexity and intractability of biofouling, their efficiency is limited. Hybrid systems are in part proposed as a sustainable antibiofouling approach to overcome the limitations of conventional biofouling mitigation technologies.

**Supplementary Information:**

The online version contains supplementary material available at 10.1007/s00284-026-04882-6.

## Introduction

In addressing recent pressing global issues centred around drinking water quality, membranes have emerged as an ideal technology to address ongoing water challenges. They function as a complementary or replacement technology in the removal of humics, particulates, and organics present in the feedwater [1]. Notwithstanding the technology’s proven success, membrane fouling remains an *Achilles’ Heel* in all membrane technology applications. Fouling negatively impacts membrane performance by shortening lifespan, reducing permeate output, increasing energy requirements, and transmembrane pressure. Over time, membrane performance will decline due to reversible and/or irreversible membrane fouling [2]. Unlike other types of fouling, biofouling is classified as a major foulant due to the ability of the microorganisms in feedwater being able to multiply, grow, and propagate forming a resultant biofilm [3]. Studies show that biofouling accounts for ~ 45% of all membrane fouling and 20–30% of operating costs in treatment plants [4, 5]. These estimations not only account for labour costs and membrane cleaning but also consider pre-treatment, downtime during cleaning period, additives/biocides used, as well as increase in energy usage at treatment plants [5].

From a material design perspective, strategies employed to mitigate biofouling include anti-microbial and anti-adhesion approaches [6]. These approaches work by either (i) inhibiting bacterial adherence (resistance-type mechanism), (ii) eliminating microorganisms approaching the membrane (release-type mechanism) or (iii) incorporating biocidal materials as a contact-killing strategy designed to inhibit growth of microorganisms (contact-type mechanism) [7]. Inhibiting bacterial adherence entails modifying the physicochemical properties of the membranes, e.g., surface roughness, hydrophobicity/hydrophilicity, electrostatic properties etc. Alternatively, attaching anti-biofouling moieties that are biomimetic on the surface membrane [8, 9]. Biocidal materials have been incorporated in/onto the membrane *via* blending, coating, photochemical grafting, or interfacial polymerisation. None of the reported strategies is sustainable, as none can eliminate bacterial attachment. Instead, the reported strategies often damage the integrity of polymeric membranes, promote resistant microbial communities, thus generating secondary pollution [10]. In the case of functionalized membranes, the contact-killing strategy is effective at eliminating bacteria; however, dead bacteria typically accumulate on the surface, forming a layer that releases extracellular polymeric substances (EPS). This ultimately results in pore clogging, therefore negatively impacting flux, selectivity, and permeation of the membranes [11]. These limitations have driven interest in environmentally friendly, targeted antifouling strategies that interrupt the biological processes responsible for biofilm formation rather than indiscriminately killing microbes.

The review discusses the phenomenon of biofouling, exploring how it occurs, its effects, and the strategies that are currently being used to eliminate it in water filtration membranes. It delves into the complex interplay between conventional mitigation strategies and the introduction of stimuli-responsive membranes, assessing their viability as a more efficient and sustainable technology in biofouling mitigation.

### Membrane Biofouling

The formation of a biofilm occurs in several stages, as depicted in Figure S1 (supplementary information). The first stage entails formation of conditioning film when the membrane is submerged water (Figure S1a). The conditioning film alters the membrane’s physicochemical properties, facilitating initial attachment of microbial cells. During the attachment step, suspended organic materials/microorganisms will either get deposited or physically adsorb loosely on the wetted membrane, forming a conditioning film [12]. The adhesion between the microorganisms and membranes is largely facilitated through physicochemical interactions, either van der Waals or electrostatic double-layer interactions. Typically, this process takes place within seconds [13]. Microorganisms’ attachment on the membranes is influenced by the following factors: (i) type of microorganisms, growth profile, availability of nutrients, population size and physiological responses of the microorganisms, (ii) properties of the membrane i.e., hydrophobic/hydrophilic, type of membrane, porosity, surface charge and roughness, (iii) characteristics of the [6]. The attachment of microorganisms is followed by the reversible attachment of planktonic microbial cells on the membrane surface (Figure S1b and S1c). The attachment is mediated by weak, non-specific forces such as Van Der Waals forces and electrostatic forces [12]. Next is the irreversible attachment of the microbial cells, which results from the secretion of EPS by the microbial cells. The biofilm cells are held in proximity by the EPS which facilitates horizontal gene transfer and cell-to-cell communication [14]. Following the irreversible phase is the maturity phase - which involves recruitment of other microbial species like fungi, protozoa, and algae (Figure S1d). Typically, during this phase, the microbial community grows, resulting in the complex heterogeneous biofilm characterised by channels and voids [15]. The channels and voids allow for nutrients and waste exchange ensuring survival and growth of the microorganisms. The final stage is the detachment and dispersal stage, where cells or clusters detach from the mature biofilm (Figure S1e). The detachment is through natural sloughing or response to changes in environmental conditions. The detached biofilm fragments then act as a seed in the formation of new biofilms on other parts of the membrane, contributing to the spread of biofouling [16].

#### Biofouling Mitigation Techniques

Several interrelated factors contribute to biofouling in membrane-based technologies. These classified under four main categories: membrane properties, operational conditions, feedwater composition and hydrodynamics and are expatiated in S 1.1.1 (supplementary information). As biofilms develop on the membrane, they create a resistance to flow that decreases the efficiency of liquid transport as described by Darcy’s law. Biofilm accumulation leads to increased hydraulic resistance, necessitating higher pressure to maintain desired flow rates [17]. Pre-treatment strategies are employed to either delay or prevent microorganisms’ adherence and are reliant on the efficiency and efficacy of the deployed biocidal materials [6]. Various pre-treatment methods, including flocculation, oxidation, ion exchange, and adsorption are very useful in slowing down fouling. Their advantages and limitations are summarised in Table S1, and further examples are discussed extensively in S1.2 (supplementary information).

### Challenges of Conventional Biofouling Mitigation Strategies

Although biofouling prevention and cleaning techniques tend to yield good results in mitigating biofouling, they still face significant challenges. For instance, the adsorption effectiveness depends on the type of foulant. Activated carbon can effectively remove hydrophobic foulants but has a low affinity for hydrophilic foulants and low-molecular-weight biopolymers, which are the main precursors of biofilm formation [18, 19]. In the case of ion exchange, it removes charged foulants but has weak to no attraction towards uncharged and weakly charged foulants, which contribute to fouling. In addition, some resins are prone to fouling, reducing their efficiency, and may require frequent replacement. In chemical cleaning, most biocides are considered harmful to the environment and pose a health risk. Highly acidic/alkaline conditions can lead to secondary environmental problems, such as harming non-target organisms or altering the ecosystem. The chemicals used can react with or corrode the membrane, compromising its structure [20]. It has been reported that mature biofilms are not readily removed by chemical cleaning, therefore requiring additional chemical cycles [11]. Despite biodispersants (natural plant extracts) being presented as safer alternatives to limit use of harsh chemicals, some of them partially degrade, causing long-term accumulation in the environment [21]. Even physical methods like air scouring cause shear forces, weakening the membrane matrix, thus reducing membrane lifespan. Additionally, the introduction of an air layer during air scouring induces crystallization on the surface, evidenced by salt crystal deposition, which unintentionally accelerates other types of fouling. Other physical methods such as electrokinetics, although eco-friendly, requires higher energy and requires a complex setup [22]. Radiation is also used to destroy microbial cells through DNA damage, effectively preventing biofilm formation. However, it has limited penetration depth, making it less effective for thick-layered biofilms. Acoustic methods using ultrasonic waves to create cavitation bubbles that detach biofouling is limited by the potential for heat generation and mechanical stress on membranes if not properly managed [23]. The operational costs of membrane fouling mitigation are very high due to the implementation of advanced pretreatment technologies and the frequent cleaning required. Researchers are leaning towards the membrane modification route as a mitigation strategy to mitigate biofouling.

### Membrane Modification Using Nanomaterials and its Limitations

To enhance the membranes’ performance, functionality, and longevity, modification is done by altering the physicochemical properties of membranes. Nanomaterials are used either to coat or functionalise membranes. Their high surface area-volume ratio, mechanical strength, stability, size, and antimicrobial properties make them suitable [24]. Detail is given in S1.3 (supplementary information). Despite their promising potential to reduce biofouling, nanoparticles have several drawbacks that hinder industrial application. Often there is a trade-off between antifouling capabilities and membrane permeability, where enhancement of one can hinder the other. There is also potential for nanomaterials to leach posing risks of secondary pollution and accumulation in environment [14]. The durability and stability are an area of concern, some degrade or lose effectiveness over time, necessitating frequent replacement. This poses a research gap in developing stable and durable nano coatings with antifouling capabilities [25]. To address some of these problems, smart stimuli-responsive membranes and biological methods can be employed as a sustainable approach to biofouling.

## Smart Stimuli-Responsive Membranes

**Fig. 1:**
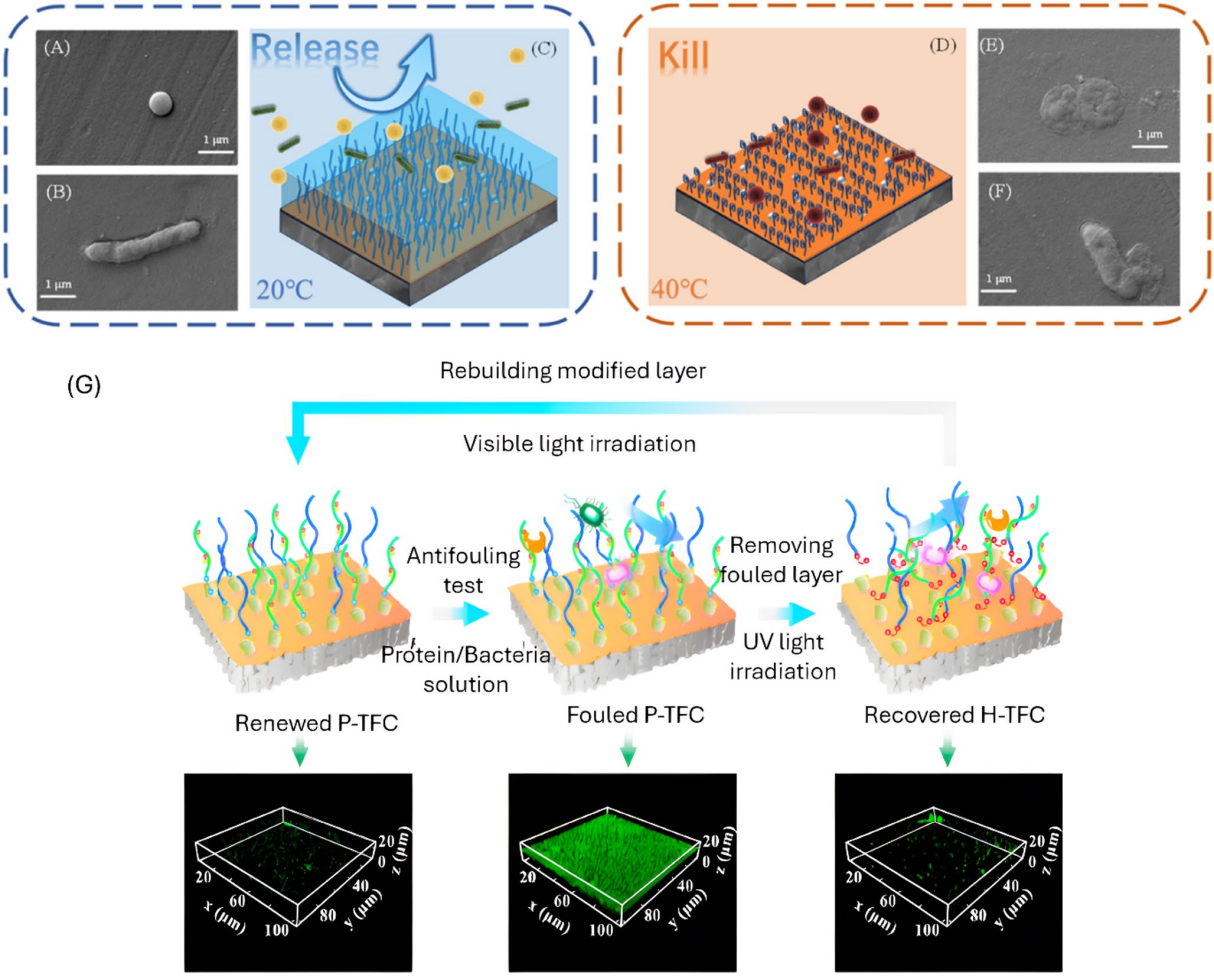
Temperature-responsive mechanism of swelling and formation of a hydration layer due to a change in temperature (**c** and **d**). Morphological characterization of S.aureus (**a** and **e**) and E.Coli (**b** and **f**) Reproduced with permission according to the terms of the CC-BY license, Copyright 2024 [27] (**g**) Light-responsive membranes depicting self-cleaning when exposed to UV light, and CLSM images of biofilm on membranes in different fouling states. Reproduced with permission according to the terms of the CC-BY license, Copyright 2020 [28]

Smart stimuli-responsive membranes have been developed to overcome challenges faced by conventional methods of biofouling mitigation they respond to a specific stimulus (i.e., pH, temperature, light, magnetic fields, biochemical signals, etc.). Polymeric membranes are made *“smart”* by coating or blending with stimuli-responsive polymers and linkers to respond to various stimuli for example for poly(ethylenimine) responds to temperature, polypyrole to light, Fe₃O₄ to electric/magnetic fields and poly(4-vinylpyridine) for pH response. Polymers like poly(N-isopropylacrylamide) are typically used in temperature-responsive membranes, they undergo changes in hydrophilicity at their lower critical solution temperature (LCST) (32 °C). Thermoresponsive membranes trap or release foulants by either heating or cooling. PNIPAAm has polymer chains that become swollen and hydrophilic below the LCST and collapse into globules, opening membrane pores above the LCST [26]. A study incorporated AgNPs in polymer; below the LCST the PNIMAAm chains swelled and formed a hydration layer **(**Fig. 1c**)**, which prevented the adhesion of bacteria. Above the LCST, the chains collapsed **(**Fig. 1d**)**, leading to the 99% bacterial inactivation by the AgNPs. The SEM examination (Fig. 1a, b, e and f**)** also revealed pronounced differences under different temperature conditions. The bacterial morphology at 40 °C exhibited indistinct boundary layers and incomplete morphological features compared to those at 20 °C, indicating temperature responsiveness in mitigating biofouling [27].

Light-responsive antibiofouling membranes use photochromic dyes or photocatalysts (TiO₂, Fe₂O₃, ZnO, etc.). Under UV or visible light, they either cleave and isomerise or generate reactive oxygen species (ROS), inactivating microbes and degrading organics in situ, enabling contactless membrane cleaning. Membranes coated with Fe₂O₃–TiO₂ had antibiofouling abilities against algae; under visible light, membranes maintained a high flux (59 L·m^− 2^·h^− 1^) after prolonged algae exposure, whereas the unmodified membrane’s flux dropped (38 L·m^− 2^·h^− 1^). The Fe₂O₃–TiO₂ surface produced ROS that inhibited *Chlorella vulgaris* attachment on the membrane [29]. Similarly, a flux recovery ratio of more than 90% was achieved after 3 cleaning cycles with a thin film membrane modified with host–guest azobenzene polymers using UV irradiation. Upon irradiation, isomerism took place; the *trans-*azobenzene translated to *cis*-configuration, and the firm connection between the Azo groups and β-cyclodextrine (which held the biofilm onto the membrane) was broken. Consequently, the biofilm was easily washed away, and the host thin film composite recovered as shown in Fig. 1g [28]. In the case of electro-responsive membranes (ERM), varying voltages are applied to the membrane matrix, which induces electro-osmotic flows and changes the local pH or oxidation states, this combined effect inhibits biofilm attachment [30]. Electrically excited polymer coatings (polyaniline/polypyroline) can reversibly swell/shrink or alter their hydrophilicity and repel foulants via Donnan effects (unequal distribution of ions across a charged membrane due to the presence of non-permeable ions) under voltage [31]. Ultrasonic membranes usually vibrate at 256 kHz when electrically stimulated, combining the generation of ROS with physical shaking. Zhao et al. (2024) demonstrated that such membranes could in-situ mitigate high-concentration oil and bacterial fouling. Under alternating current, the ultrasound not only dislodged foulants from the membrane surface but also produced ROS, providing a bacterial-organic-inorganic antifouling effect simultaneously [32].

pH-responsive membranes (PRMs) undergo structural/chemical changes in response to pH, making them highly effective in preventing/minimizing biofouling [33]. Polymers with ionizable groups that respond to pH changes due to protonation or deprotonation are preferred materials as they facilitate the disruption of the biofilm matrix or the release of antifouling agents [34]. PRMs respond to changes in pH using various mechanisms some undergo shrinking/expanding at different pH. For instance, polymers containing a weak acid (-COOH) group expand at high pH and collapse at low pH, while basic polymers (-NH_2_) do the opposite. Polyethyl acrylic acid chains usually swell in basic media due to deprotonation, narrowing the membrane pores and repelling foulants, however, they collapse in acidic media. Conversely, poly-2-dimethylaminoethyl methacrylate (PDMAEMA) or poly-4-vinylpyridine (PVP) protonate in acid media and expand but collapse in basic media, resulting in pore modulation which regulates the rejection of foulants [35]. PRMs also alter their hydrophilicity in response to pH changes, enhancing antifouling characteristics, improving filtration performance, and reducing foulant accumulation during processes like reverse osmosis and ultrafiltration [34]. pH-responsive materials like PDMAEMA, polymethacrylic acid, PAA, alginate, polyethyleneimine, and poly(L-lysine) can be incorporated to the membrane matrix and enhance anti-fouling properties by altering hydrophilicity in response to pH changes. This modification significantly reduces membrane fouling, as demonstrated by improved flux decay rates of 7.6% against 15.6% and flux recovery ratios of 93.2% and 57.8% in modified NF membranes compared to virgin membranes [34, 36].

pH-sensitive materials typically contain carboxyl, amine, or zwitterionic groups that undergoes ionization changes with pH changes. Surface charge reversal may occur due to the alteration of the zeta potential. Amino-based polymers become strongly positively charged at a pH below the polyelectrolyte’s pKa and repel positively charged biofoulants while repelling anionic foulants at high pH [37]. For example, polydopamine, tannic acid and chitosan formed multilayer coatings that release allicin biocides in response to pH changes, effectively inhibiting the growth of bacteria by more than 60% [38]. Elsewhere, 3-aminophenyl boronic acid and trimesoyl chloride were grafted on the membrane surface exploiting the diol-groups, the biocides were released under acidic conditions due to the pH-responsive nature of boronate ester complexes [39].

### Shortfalls of Stimulus-Responsive Membranes

Although stimuli-responsive membranes are gaining popularity in research, their industrial applications remain limited. Some researchers have worked on scaling up to the model phase, but the long-term stability remains worrisome, especially under changing environmental conditions. Repeated swelling/deswelling or structural rearrangements may lead to polymer fatigue and degradation. Maintaining ideal conditions, such as an ideal pH range or temperature, at which these stimuli-responsive membranes can respond effectively, also presents a challenge; sometimes fouling occurs in areas of the membrane where the pH-responsive system is not sufficiently triggered due to localized pH variation and insufficient activation. Achieving precise control over timing and number of biocides being released is not easy, it necessitates precise material engineering to develop systems that react to pH changes consistently and reversibly, making membrane fabrication more difficult. Ultimately, an increase in capital and operational costs due to the need for frequent membrane replacement, automation, real-time monitoring, and tighter process control becomes inevitable.

## Biological Processes of Biofouling Mitigation

Biological approaches to biofouling mitigation exploit natural biological processes to interrupt the growth of biofoulants on membrane surfaces. Such methods include quorum quenching, energy coupling, bacteriophage etc., this review however, will focus exclusively on enzymes-based methods.

### Enzymes in Biological Cleaning of Biofilms

**Fig. 2:**
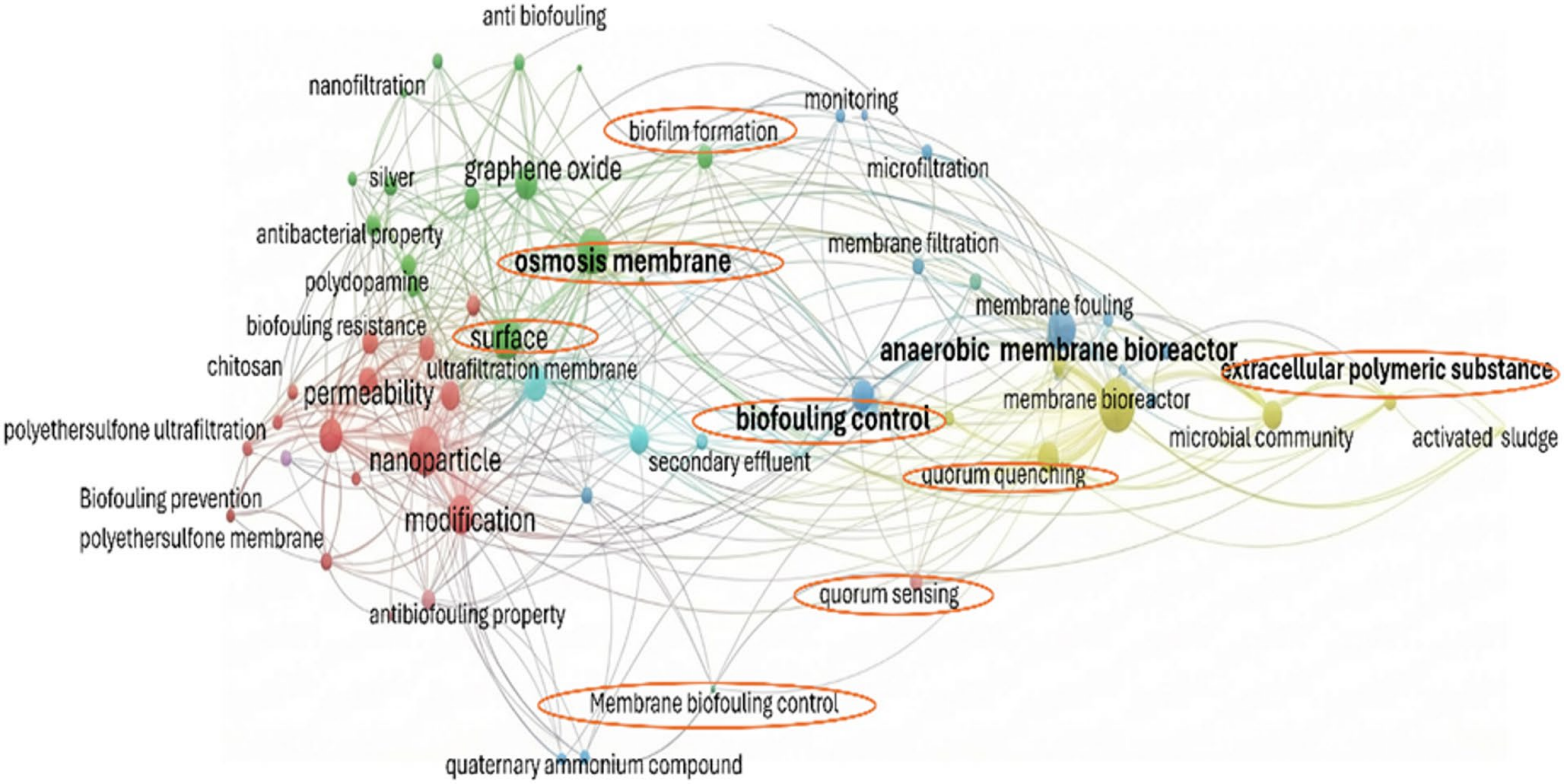
Bibliometric network analysis showing co-occurrence keyword network analysis for membrane biofouling. Reproduced with permission according to the terms of the CC-BY license, Copyright 2024 [40]

**Fig. 3:**
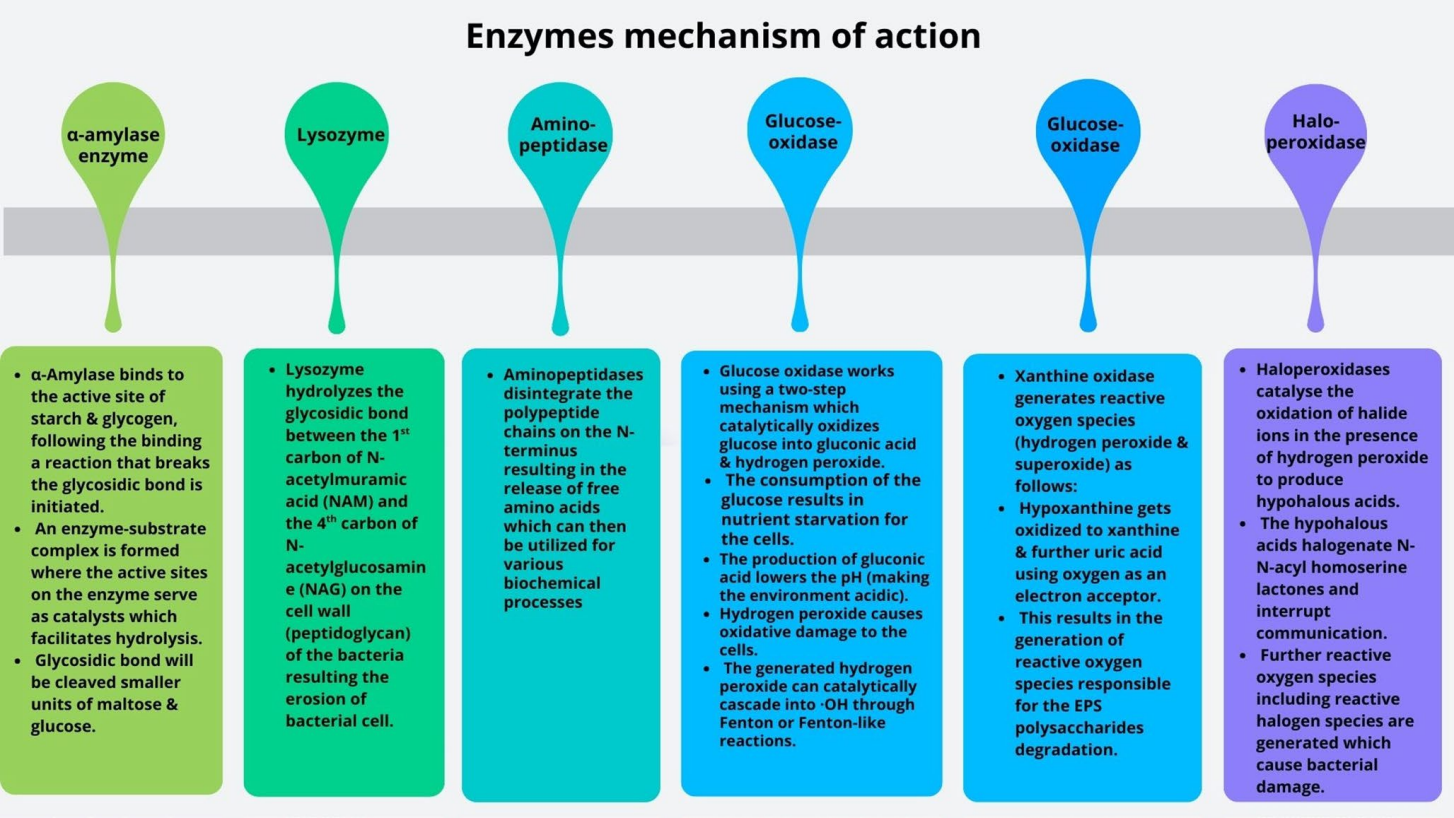
Schematic summary of anticipated enzymes’ mechanism of action in biofouling mitigation

A recent bibliometric network analysis (Fig. 2) analysing research trends on membrane fouling over the past 10 years showed that most of the research conducted on biofouling has been mainly on reverse osmosis [40]. From the bibliometric network analysis, it is evident that the use of enzymes has been an under-explored. Although under-explored for this purpose enzymes selectively degrade key biofilm components [41]. They disrupt and penetrate the biofilm, thus reducing bacterial cell viability and altering the surface morphology of biofilms [42]. Various enzymes have been used to mitigate biofouling and are summarized in Table S3 while Fig. 3 illustrates the mechanism of action of select enzymes.

Enzyme-based biofouling control methods appear to be a better alternative to conventional physical or chemical cleaning, owing to their high specificity, gentle action on membrane materials, and ability to degrade biofilm matrix components (EPS, proteins, polysaccharides) rather than detach loose deposits [43]. Experimental evidence shows that enzymes can reduce attached biomass by up to 70% and restore membrane surface characteristics (hydrophobicity, roughness) to near-pristine levels [44]. However, economic constraints, the need for optimized enzyme cocktails, and limited long-term real-world data currently hinder widespread adoption. Nonetheless, when combined with immobilisation or stimuli-responsive enzyme-based strategies, they may represent the next generation of sustainable, high-efficiency fouling control, capable of addressing complex biofilm formation and reducing membrane degradation over time [45].Typically, in the early stages of fouling, proteolytic enzymes, including proteinase K, trypsin-EDTA and papain are highly effective against protein-dominated biofilms. Proteinase K achieved over 91% reduction in *S-aureus* and E-*coli* biofilms on polystyrene membrane surfaces [46] highlighting its potential to degrade protein-based adhesion layers that initiate fouling. Trypsin–EDTA contributed by breaking down proteins into smaller peptides and chelating divalent cations, resulting in up to 71% biofilm reduction when used in enzyme cocktails [44]. Papain, a broad-spectrum cysteine protease, also offered significant biofilm removal and inhibition, achieving up to 59% reduction against *Listeria monocytogenes* at relatively low concentrations [47]. However, despite their high catalytic activity, proteases are constrained by factors such as cost, susceptibility to denaturation at extreme pH or temperature, and sensitivity to metal ions commonly found in wastewater streams. Therefore, these enzymes are most effective for preventing or treating early-stage fouling rather than for removing mature, complex biofilms [43].

In contrast, polysaccharide-degrading enzymes are most applicable for mature, structurally robust biofilms, particularly those dominated by *P. aeruginosa*, which produce EPS-rich matrices. α-Amylase demonstrates high efficacy against polysaccharide scaffolds, achieving 92–97% removal of preformed biofilms on RO membranes [48]. By focusing on mannosidic bonds essential to EPS integrity, β-Mannosidase has been shown to perform better than several other enzymes under similar conditions [44]. Endoglucanase also reduced *S. aureus* and *P. aeruginosa* biofilms by 76.8% and 61.7%, respectively, by rupturing cellulose-based or mixed polysaccharide matrices [49]. Among this class, alginate lyase stands out for both performance and stability. Alginate is a dominant EPS polysaccharide in *P. aeruginosa* biofilms as a result alginate lyase directly weakens the structural matrix and shows strong longevity, retaining 80% activity after 21 days [50]. Its application in UF/MF membrane systems further resulted in an 82% reduction in foulant resistance, underscoring its role as a high-value enzyme for severe biofouling scenarios [51]. Collectively, these results indicate that polysaccharide-targeting enzymes are essential for degrading the structural matrix of mature biofilms and are therefore well-positioned to clean or rehabilitate heavily fouled membranes.

Enzymes that target cell walls, such as lysozyme, exhibit rapid antimicrobial activity and are particularly effective during the early stages of biofilm formation. Lysozyme can reduce viable bacterial counts by more than 80% on polystyrene surfaces, making it effective against Gram-positive bacteria [52]. However, it is significantly less effective against Gram-negative bacteria and has a limited impact on established biofilms rich in EPS. Additionally, lysozyme’s scalability for water treatment applications is limited by its high production cost and susceptibility to salt interference [53]. Quorum-quenching enzymes, exemplified by acylase, represent a unique preventive approach. Specifically, by degrading signalling molecules essential for bacterial communication, acylase reduces EPS production and alters biofilm morphology, thereby delaying biofilm establishment. Reported biofilm reductions range from 20 to 24% on RO membranes to 60–73% on polystyrene [54]. These enzymes are nonetheless very promising as long-term antifouling solutions, even though they are insufficient as stand-alone, especially when immobilized or incorporated into stimuli-responsive systems that enable controlled activation during filtration cycles [55].

It is important to consider how biofilms grow, what they are composed of, and how the system operates when selecting an enzyme for membrane cleaning. Enzymes that break down certain sugars, especially alginate lyase and β-mannosidase, work best and last longest against complex, mixed groups of bacteria that form tough films in standard water treatment systems. In comparison, enzymes that break down proteins are better for early or simple protein buildup. Also, adding enzymes that can interfere with bacterial communication into special membrane systems offers clear benefits. These results show that using the right mix of enzymes, along with careful methods to keep them in place, is key to achieving the best cleaning results and saving money in these systems [44].

## Proposed Solution in Biofouling Mitigation

Given the limitations of conventional biofouling mitigation methods and the stimuli-responsive membranes, there is an opportunity to fabricate hybrid membranes using crosslinking to improve their mechanical and chemical stability. Enzymes can be effectively incorporated into nanomaterials to reduce biofouling, leveraging their unique properties to enhance performance in such hybrid applications. Combining enzymes with nanomaterials improves stability and efficiency and adds new functions that can contribute to biofouling mitigation [56]. To date, research has focused on either enzymes alone or nanomaterials alone and rarely combined. Lin et al. (2021) investigated a facile renewable anti-biofouling layer strategy wherein smart enzymatic nanomaterials self-assemble onto a commercial membrane surface to construct a pH-triggered, releasable, and regenerable anti-biofouling system. The proteinase-K-functionalized, PEGylated silica (SPK) nanoparticles, deployed in the study resulted in improved membrane properties, enhanced performance, and extended membrane lifespan [57].

As discussed earlier, when fouling occurs, biofoulants release EPS and toxins from their metabolic processes, altering the membrane pH. Therefore, it is more effective to employ techniques and materials that degrade biofoulants rather than those that merely repel them. The integration of immobilised enzymes with GQDs represents a compelling advancement in pH-triggered biofouling mitigation. This hybrid approach leverages the complementary strengths of both enzymatic catalysis and nanomaterial properties to create intelligent, responsive systems capable of targeting biofilm components on demand. A relatively underexplored nanomaterial with significant potential as a nanocarrier for enzyme immobilization is graphene oxide quantum dots (GQDs); they offer excellent surface area, biocompatibility, tunable functional groups, and exceptional dispersibility. Enzymes such as laccase, glucose oxidase, and protease retain high catalytic activity when immobilized on GQDs and have demonstrated efficacy in degrading biofilm components, including polysaccharides, proteins, and extracellular DNA [45, 58]. The abundance of -COOH and -OH functional groups on GQDs allow strong bonding to membranes and polymers, thereby reducing the risk of leaching and ensuring long-term membrane performance. They allow integration with an imaginative trigger due to their photoresponsive and pH-sensitive nature [59]. Incorporating GQDs and enzymes provides strong biofilm resistance and self-cleaning. Still, polymers will also be needed to support the structural matrix, tune stimulus response, and provide anchoring sites for GQDs and enzymes, thereby enhancing stability. Although pH-triggered enzyme exposure has mainly been demonstrated in GO/GQD/chitosan nanochannel sensors that respond to pH shifts via fluorescence changes and in drug delivery, the principle could be applied to antifouling smart membranes [60, 61]. This enzymatic system can be coupled with pH-responsive polymers or linkers, enabling smart, on-demand enzyme activation. A pH-sensitive polymer could bind the GQD–enzyme complex and release the enzyme in low-pH microenvironments typical of growing biofilms, enabling localized enzymatic action [62]. The pH-sensitive polymers in enzyme-GQD hybrid systems undergo reversible changes in response to local pH variations. At neutral pH, these polymers are compact, tightly binding the enzyme-GQD complex to the membrane and suppressing enzymatic activity. When they encounter acidic microenvironments, the polymers swell and release the enzymes, which then degrade biofilm components. This localized release ensures that enzymatic activity is concentrated where it is most needed. This pH-responsive mechanism allows repeated cycles of biofouling mitigation. Once biofilm components are degraded, the membrane’s pH normalizes, and the polymer returns to its compacted state, re-binding the enzyme-GQD complex. This process helps maintain enzyme longevity and minimizes unnecessary degradation of membrane components, enabling the same enzyme-GQD units to be activated multiple times throughout the membrane’s operational life [61].

The enzymatic degradation of biofilm components occurs through multiple parallel pathways, reflecting the diverse composition of biofilm matrices. Biofilms typically contain significant amounts of polysaccharides, including both homopolysaccharides and heteropolysaccharides, which are carbohydrate components of lipopolysaccharides. The degradation of these polysaccharides can be facilitated by endoglycanases that cleave internal glycosidic bonds or exoglycanases that sequentially remove saccharide units from the ends of the polymer. The resulting oligomeric and monomeric sugars are more soluble and more readily accessible for further degradation or transport out of the biofilm region, disrupting the hydrated, gel-like polysaccharide matrix that provides structural integrity to the biofilm [63–65]. Protein components are essential constituents of biofilms, comprising up to 50% of the dry mass in specific systems. Proteases immobilized on GQDs catalyze the hydrolysis of peptide bonds, resulting in the formation of oligopeptides and free amino acids. The degradation of extracellular proteins, such as biofilm-associated adhesions that mediate cell-cell interactions, disrupts biofilm structure and weakens intercellular adhesion. Furthermore, numerous biofilm-associated proteins exhibit enzymatic activity or function as cofactors in biofilm metabolism; their degradation therefore impairs the biofilm’s metabolic capacity and overall viability [64].

Extracellular DNA (eDNA) is another critical component of biofilms, serving both structural and regulatory roles. Recent research has shown that eDNA contributes significantly to the mechanical properties of biofilms and provides essential electron-transfer pathways in those composed of electroactive species. Nucleases immobilized on GQDs or localized with other enzymatic activities can break phosphodiester bonds in eDNA, fragmenting the DNA backbone and diminishing its ability to maintain biofilm structure. The combined enzymatic degradation of polysaccharides, proteins, and DNA simultaneously targets multiple structural elements of the biofilm, creating synergistic effects that are more effective than the use of single-enzyme systems [66].

### Technical Challenges and Mitigation Strategies

#### Enzyme Leaching and Retention

Enzyme leaching poses a significant challenge for enzyme-based antifouling systems. Although GQDs functional groups and polymer crosslinking provide strong binding, thermodynamic equilibrium often leads to some enzyme desorption [67]. The risk of leaching increases in the activated state of pH-responsive systems, where polymer swelling enhances enzyme accessibility and may reduce binding affinity due to conformational changes. This loss of enzymes can decrease system effectiveness and potentially cause nonspecific degradation of components or the production of harmful byproducts. To minimize enzyme leaching effectively, a comprehensive approach is essential. Covalent immobilization is a promising method for creating stable bonds between enzyme lysine residues and GQDs using techniques such as EDC/NHS chemistry and glutaraldehyde crosslinking. These robust linkages are resistant to thermal motion and osmotic pressure, reducing desorption risk. Another effective strategy is to use high-crosslink-density polymer matrices, which limit enzyme mobility and further reduce leaching rates. Additionally, multivalent immobilization, which involves multiple attachment points, enhances enzyme stability. This ensures that even if some connections break, the remaining ones can still secure the enzyme. Combining these strategies can improve enzyme stability and performance in various applications, leading to exciting advancements in the field [68, 69].

#### Enzymatic Stability and Denaturation

Enzymes immobilized on nanomaterial surfaces often face stresses that reduce their stability compared to those in solution. This immobilization can lead to partial unfolding or conformational changes at the enzyme-nanomaterial interface, thereby disrupting the active-site geometry. Also, the proximity of enzymes on high-surface-area nanocarriers may also result in unfavourable enzyme-enzyme interactions, such as aggregation [67]. The hydrophobic nature of graphene surfaces can promote the unfolding of hydrophobic protein cores, while covalent crosslinking during immobilization can introduce mechanical stress on the enzyme’s tertiary structure. The pH-responsive activation and deactivation cycles in the proposed system introduce stability challenges. The repeated swelling and deswelling of the polymeric matrix subject immobilized enzymes to mechanical stresses, which can degrade their structure. These conformational changes create shear forces that may disrupt the enzyme’s tertiary structure. Additionally, the low-pH conditions that trigger polymer expansion can denature enzymes, especially those with limited acid stability. Prolonged exposure to acidic environments can lead to protonation of ionizable residues and hydrolysis of sensitive covalent bonds, such as disulfide bridges and thioester linkages [70].

Stability of immobilized enzymes requires multifaceted approaches. Protective coatings around individual enzyme molecules using biocompatible polymers (such as polyethylene glycol or polysaccharides) buffer the enzyme microenvironment and reduce exposure to pH extremes [71]. The rational selection of buffer systems and the inclusion of osmolytes (such as sorbitol or glycerol) provide osmotic protection and reduce the degree of dehydration enzymes experience on nanomaterial surfaces. Optimizing immobilization chemistry to minimize protein unfolding and denaturation by selecting mild crosslinking methods, low crosslinking densities, and enzyme-friendly spacer molecules reduces immobilization-induced stress. Periodic assessment of enzyme activity using standard kinetic assays establishes baseline activity. It quantifies activity loss over operational time, enabling the prediction of the enzyme-GQDs system’s lifespan and planning regeneration or replacement cycles.

#### Mass Transfer Limitations

The enzymatic degradation of biofilm components is fundamentally limited by the rate at which substrate molecules (biofilm polymers) can diffuse from the biofilm matrix to the enzyme active sites. In solution-phase reactions, mass transfer limitations are generally minor as turbulent flow and convection rapidly replenish depleted substrates. However, within the complex, heterogeneous environment of a membrane biofilm, mass transfer represents a critical rate-limiting step. The dense polymer matrix of biofilms restricts diffusion rates to values substantially lower than in bulk solution, with effective diffusion coefficients often reduced by factors of 10–100 compared to aqueous solution. The presence of multiple enzyme types competing for limited substrate creates additional kinetic complexity and may lead to substrate depletion near enzyme active sites [72, 73]. Strategies to mitigate mass transfer limitations must address the fundamental diffusional barriers within biofilms. The spatial distribution of enzymatic activity directly impacts mass transfer efficacy; distributed enzyme systems that span the biofilm volume provide more localized enzymatic action and reduce the maximum diffusion distances for substrate molecules. The incorporation of GQD-enzyme complexes throughout the membrane depth, rather than exclusively at the biofilm-membrane interface, improves accessibility of internal biofilm regions to enzymatic degradation. The sequential or staged activation of different enzyme types may optimize substrate utilization by preventing competition for limited substrates. For example, exoglycosidases that generate smaller oligosaccharide products might be activated before endoglycosidases, reducing diffusion distances for intermediate products [67].

#### Enzyme Activity Control and Specificity

The reversible activation and deactivation of enzyme-GQDs systems require precise control over the microenvironmental conditions that trigger conformational changes in the polymer [74]. Even slight variations in pH can significantly alter the degree of polymer expansion and the resulting enzyme activity, posing challenges for maintaining optimal enzymatic activity while minimizing excessive non-specific degradation. It is essential to carefully tune the pH threshold for enzyme release to differentiate between acidic microenvironments associated with biofilms and transient pH changes in the bulk solution that should not initiate enzyme activation. Compartmentalizing enzymatic activity within biofilm microenvironments through pH-triggered activation greatly enhances specificity by limiting enzyme exposure to bulk membrane regions that maintain a neutral pH. Furthermore, the thoughtful selection of enzyme substrates and catalytic mechanisms that exploit the unique characteristics of biofilm components can improve specificity. For instance, enzymes that target cross-linked biofilm matrix components, which are absent from membrane structures, can facilitate selective biofilm degradation while minimizing potential damage to membranes [74, 75].

The proposed solution of using the pH-responsive enzymes and GQDs requires higher upfront material and synthesis costs due to advanced nanomaterials and immobilization technologies, but enhances the antimicrobial and antibiofouling membrane surface properties, which offer a long-term performance and reduce operational costs. This is achieved through the reduced microbial adhesion, enhanced hydrophilicity and flux retention, and inhibition of biofilm development under filtration conditions as a result of GQDs incorporation into membrane materials, leading to better sustained performance and fewer cleaning cycles [76]. On the other hand, the enzymes effectively target the biological mechanisms of biofilm formation and can delay or reduce fouling without harsh chemical cleaning, potentially lowering maintenance costs and energy use over time [41].

## Conclusion

This review examined biofouling, conventional mitigation strategies, as well as stimuli-responsive strategies of mitigating biofouling in trying to address the need for sustainable methods of biofouling control. The review proposes the use of hybrid systems that use one or more techniques or materials, leveraging the strengths of each. Stimuli (especially pH) responsive enzymes and responsive materials offer a robust, sustainable tool against biofouling. Despite existing studies on enzymes, nanomaterials (GQDs), and responsive polymers in membrane biofouling, a research gap remains in integrating these materials, which is anticipated to improve durability and lifespan, as well as scalability. Future research should address these gaps by testing the proposed materials at a lab scale and also scale up to plant application using the following recommendations:

- The pH-responsive materials should be designed to operate over a wide range of pH.
- For a more effective and multifaceted approach to mitigating biofouling, a combination of pH-triggered materials and biocides/antimicrobial enzymes can be used, with these enzymes/biocides released in response to pH changes.
- Applying approaches like the layer-by-layer method of membrane coating, which allows for localised release of biocidal agents to areas prone to fouling.
- Establish protocols for periodic testing of pH-triggered systems and for adjusting their design or operational conditions as needed. Regular evaluation ensures that systems are functioning optimally and helps detect issues early.
- Integration of feedback loops, where the release of biocidal agents is controlled based on the real-time detection of fouling or pH changes. As the systems are developed, it is necessary to ensure they can be easily scaled up for industrial use.

## Supplementary Information

Below is the link to the electronic supplementary material.

Supplementary Material 1

## Data Availability

No datasets were generated or analysed during the current study.
