# Supplementary material for: Membrane Biofouling: Current Understanding and Future Perspectives- An Overview
Source: Curr Microbiol. 2026 Apr 14;83(6):299. doi: 10.1007/s00284-026-04882-6 (PMC13079486; doi:10.1007/s00284-026-04882-6)
Supplement: Supplementary file 1 — Supplementary Material 1 [file 284_2026_4882_MOESM1_ESM.docx]

**Membrane biofouling: Current understanding and future perspectives- An overview**

N. Phillip, A. A Muleja, M.M Motsa, B.B Mamba, T.T.I Nkambule, C.S. Tshangana*

*Institute for Nanotechnology and Water Sustainability, College of Science, Engineering and Technology, University of South Africa, Johannesburg 1709, South Africa.*

***Corresponding author:** [tshansc@unisa.ac.za](mailto:tshansc@unisa.ac.za)

- 1. **Membrane biofouling**


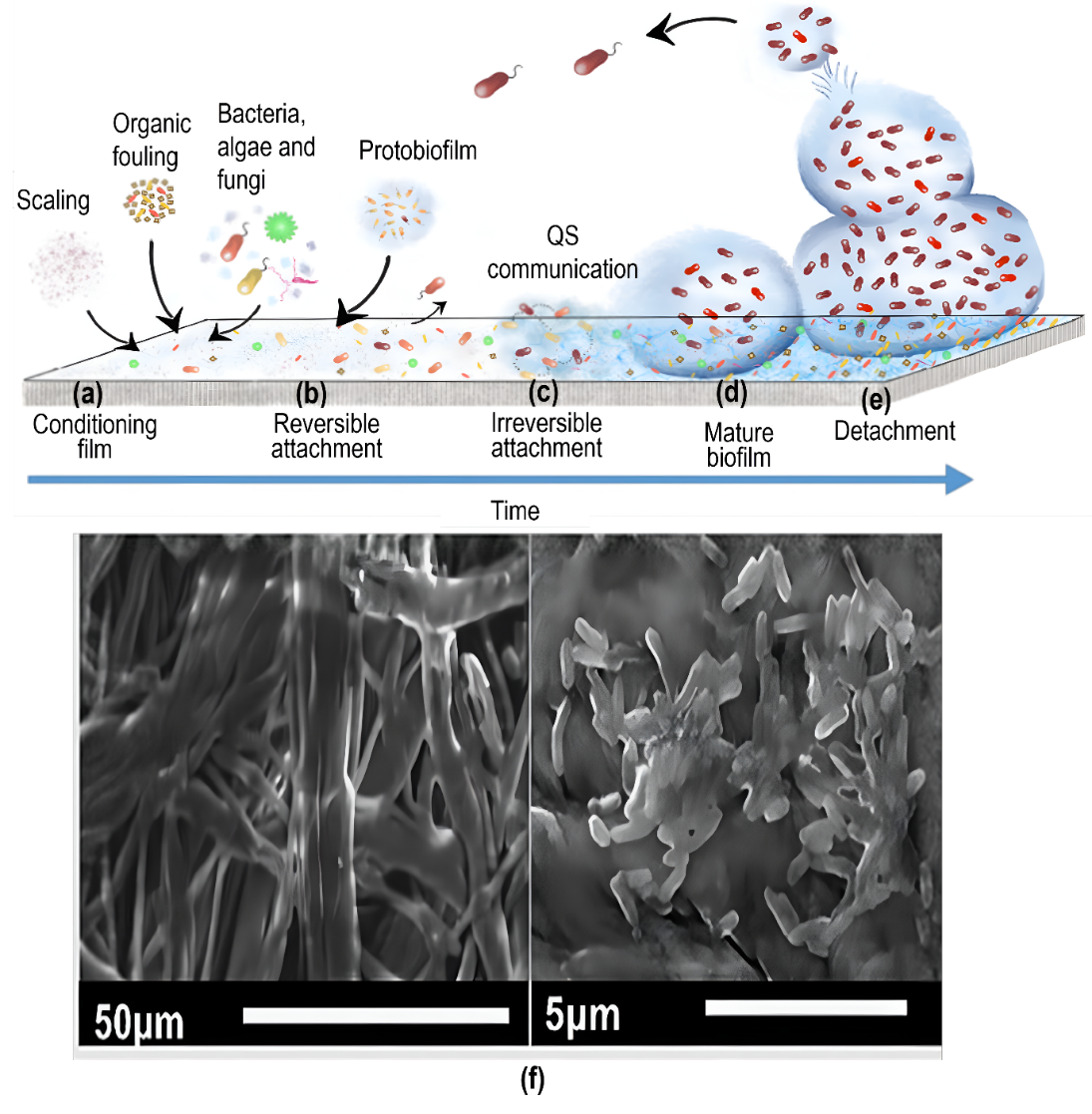


**Figure S1**: Stage-by-stage development of biofilm on the membrane surface depicting the various growth (a) conditioning film, (b) reversible attachment, (c) irreversible attachment, (d) mature biofilm, (e) detachment, *Reproduced with permission according to the terms of the CC-BY license, Copyright 2021* [1].

- - 1. **Factors influencing biofouling in membranes**

### **Membrane properties**

Membrane physicochemical properties play a crucial role in microbial adhesion and the growth of biofilm and the extent of biofouling in membranes [2]. Rougher membrane surfaces (Ra>50nm) typically encourage higher levels of initial microbial adhesion, with smooth surfaces being 35% less affected by biofouling [3]. Membrane surface roughness enhances cell aggregation and provides more sites for bacterial colonisation. This is attributable to increased available surface area and concealed spaces open to the settling of microorganisms [4]. Membrane hydrophobicity also influences biofouling through hydrophobic interactions, wherein biofoulants are drawn to hydrophobic membranes. Conversely, hydrophilic membrane surfaces show less adherence due to the water layer that prevents direct contact between microorganisms and the membrane surface [5]. Other membrane properties which influence biofouling include membrane surface charge, membrane material composition and porosity. Membrane surface charge influences the deposition of charged biofoulants and the interaction between the membrane and microbial cells. Slightly negatively charged or neutral membrane surfaces have lower fouling susceptibility [6]. The material used to fabricate membranes significantly impacts the fouling behaviour by controlling the physical and chemical properties of the membrane. Different materials used to fabricate membranes display differing affinities for microbial attachment, which affects overall biofouling behaviour. Polymeric and ceramic membranes possess different properties, which give them distinct behaviours towards biofouling. Ceramic membranes generally show superior mechanical and chemical stability, which leads to enhanced biofouling resistance as compared to polymeric membranes [7,8].

Membrane porosity also influences both microbial adhesion and mass transport. The porosity of membranes needs to be optimised to ensure excellent performance of membranes. The effect of pore size on ceramic membrane was investigated, and mid-range pore sizes of 7µm were found to have the best performance balancing biofilm resistance and flux [9]. On the other hand, membranes with high uniform porosity of 80% also demonstrated better performance and biofouling resistance, indicating that the membrane pore volume and uniformity have a great influence on the biofouling potential of membranes [10]. In addition, membrane module design also influences biofouling as it affects the flow patterns of the water, the shear patterns, and the residence time of the feedwater [11]. A membrane module is a structural component that houses and supports a membrane for separation or filtration purposes. Common designs include spiral-wound and hollow fibre configurations, which maximise surface area while minimising pressure drops [12]. Spiral wound or plate and frame designs shown favour crossflow dynamics wherein water flows tangentially to the membrane surface, which helps to sweep away foulants. While in a dead-end flow system, the flow is perpendicular to the surface of the membrane which can promote fouling since there are minimal forces to remove foulants [13]. Module geometry and spacing can also influence biofouling as they influence the flow dynamics, wall shear stress, pressure drop, and interfacial interactions. Space between the individual membranes in a module can influence biofilm build-up; for example, too tight a space can cause inadequate flow or shear forces to disrupt biofilm formation and growth. Poorly designed spacers can cause stagnant flow, which promotes biofilm formation [14].

### **Operational conditions**

The operational parameters of MBTs influence the development and growth of biofilms. These parameters include temperature, pressure, pH, ionic strength, and the frequency of membrane cleaning. Temperatures above 35°C typically promote increased microbial activity and growth, resulting in accelerated biofilm formation. Optimal temperature ranges for microbial growth can vary between 15°C- 70°C, depending on the specific microorganisms present in the system [15,16]. Typically, elevated temperatures reduce the viscosity of the feed and enhance the diffusivity; this phenomenon enhances the rate of transport of foulants carried from the membrane surface and returned to the bulk solution [17]. Feedwater's pH level contributes significantly to the likelihood of biofouling. Biofouling is particularly likely to occur at neutral to slightly alkaline pH levels, typically around pH 7 to 7.4, where protein adsorption responses are heightened [18]. Additionally, feedwater flow rate influences the fluid velocities and the movement of microorganisms by causing shear stress on the filtration membrane surface. Increased shear forces produced by higher flow rates can loosen adherent biofilm, reducing the consequences of fouling [19]. One of the important factors in ensuring effective filtration is the operating pressure, which directly affects flux according to Darcy’s law in **Equation S1** [20]**.** Applied pressure significantly influences membrane filtration by increasing flux, reducing intrinsic membrane resistance, and determining critical flux levels. Higher operating pressures can generate shear forces that may delay initial attachment, detach, and disrupt biofilm fragments, but can also compress the biofilm structure, increasing fouling in the long run [21]. Ceramic membranes were tested using the internal and external suction method by who observed that maintaining operational pressures below critical levels is vital for sustaining flux and minimising fouling [20]. The critical pressure (in this case, between 4-5kPa) was defined as the pressure level at which the membrane began to experience fouling, leading to a decrease in flux. The maximum flux that can be attained in membrane filtration without causing a significant drop in flux due to excessive fouling is called critical flux [20,22].

$\mathbf{J}\left( \mathbf{t} \right)\mathbf{=a}\frac{\boldsymbol{\Delta P}}{\boldsymbol{\mu}\mathbf{R}_{\mathbf{m0}}}\mathbf{e}^{\mathbf{-bt}}\mathbf{+c}$ **(Eq S1)**

*where J(t) is the membrane flux (L·h^−1^·m^−2^) at different times, ΔP the applied filtration pressure (kPa), μ the hydrodynamic viscosity (Pa·s), and a, b and c the experimental parameters, obtained by curve-fitting the experimental data [20].*

Controlling biofouling requires routine membrane cleaning; system performance as a whole and biofilm maturation depend on the frequency of the cleaning cycle. Regular cleaning can help decrease the buildup of biomass, but it must be weighed against the effects and expenses of operation [23]. Zhang et al. (2024) reported that increasing the frequency of cleaning using free ammonia prolonged the membrane lifespan. This was attributed to free ammonia being able to effectively degrade proteins, polysaccharides and adenosine triphosphate [24].

### **Feedwater composition**

The feedwater usually contains microorganisms, nutrients, ions, and organic matter which are essential for the adhesion and growth of biofilms. Dissolved organic carbon and other organic compounds present in the feedwater provide nutrients for the microbes, which promote the growth of biofilms. Certain types of organic foulants, such as NOM have higher adhesive properties and provide attachment sites for microbial cells, promoting initial microbial adhesion [25]. Diverse microbial populations cause uneven growth rates of the biofilm, which can impact the fouling dynamics, as each type of foulant has its own growth rate and adhesion process [26]. These microorganisms depend on the nutrients for survival and growth; therefore, the availability of nutrients in the feedwater. Nitrogen, phosphorus and potassium in feedwater specifically promote increased microbial activity and the formation and growth of biofilms, as defined in **Equation S2** [25]. The ionic strength of feedwater also affects electrostatic interactions, altering how microorganisms interact with the membrane surface and, eventually, microbial attachment as well as biofilm formation. Higher ionic strength enhances the stability of microbial adhesion to surfaces [27].

$\boldsymbol{\mu}=\frac{\boldsymbol{\mu}_{max}\boldsymbol{S}}{\boldsymbol{K}_{\boldsymbol{s}}+\boldsymbol{S}}$  **(Eq S2)**

*where:* $\boldsymbol{\mu}_{\boldsymbol{}}$ *is the specific growth rate (h^−1^),* $\boldsymbol{\mu}_{\boldsymbol{max}}$ *maximum specific growth rate S substrate (nutrient) concentration, K_S_ is the half-saturation constant (substrate concentration at which* $\boldsymbol{\mu=}\frac{\boldsymbol{\mu}_{\boldsymbol{max}}}{\boldsymbol{2}}$*).*

### **Hydrodynamics**

Hydrodynamics (flow velocity, shear stress, and nutrient transport) significantly influence biofouling through various mechanisms [28]. High flow velocities, typically in the range of 0.1-0.3 m/s, are favoured to low velocities as they generally cause strong shear forces that sweep away foulants from the membrane surface, preventing them from having time to attach onto membrane surfaces, reducing fouling [29]. Therefore, regulation of flow velocity is very important in controlling biofouling. The hydrodynamic conditions around membranes can be critical in dictating fouling behaviour. Laminar flow (typically Re < 2000) is often associated with increased biofouling as opposed to turbulent flow regimes (Re > 4000), which typically disrupt microbial attachment and promote better mass transfer, helping to mitigate fouling [30,31]. Shear stress is a vital parameter influencing membrane performance and biofouling. Optimal shear stress levels can hinder biofilm growth by dislodging attached microorganisms and preventing stable adherence [32]. Wall shear stress plays a critical role in this process, with studies demonstrating that higher shear stress values (approximately 5.6 Pa) result in significantly thinner biofilms compared to lower shear stress conditions (0.2 Pa). For microalgae biofilms, shear stress levels of 1.0, 6.5, and 11.0 mPa have been investigated, with higher values (11.0 mPa) significantly increasing biofilm cohesion and allowing them to grow thicker and produce more biomass as a biological response to resist the applied stress [33,34].However, insufficient shear stress may allow for significant fouling to develop over time, presenting a challenge for operational stability [14].

**2.1 Biofouling mitigation techniques**

Various pre-treatment methods, including flocculation, oxidation, ion exchange, and adsorption, have been studied and are very useful in slowing down Their advantages and limitations are summarised in **Table S1**.

- 1. **Pre-treatment of feedwater as biofouling mitigation strategies**

Adsorption reduces biofouling by removing phosphorus and organic matter, which serve as sources of nutrients for bacteria. Activated carbon is the most widely used adsorbent. In a study using powdered activated carbon (PAC), improvements in flux rates, 78% removal of chemical oxygen demand (COD) and 97% removal of suspended solids were observed, all of which are attributed to bacterial growth inhibition as the nutrients and COD are reduced [35]. In other instances, adsorption can also be deployed as a pre-treatment step using activated carbon beds (e.g., granular activated carbon, GAC) or combined GAC–PAC contactors upstream of membranes to strip biodegradable organic fractions (AOC/biopolymers) and limit initial biofilm formation. A 2024 experimental study coupling GAC and PAC with a membrane bioreactor reported 11.713.6% lower total (especially cake-layer) filtration resistance versus controls, a 15–24% reduction in fouling rate at critical-flux steps, and near-complete removal of organic matter with 99% total phosphorus removal; collectively translating to slower TMP rise and improved run length. Another study also reported that with an NF system with PAC dosed operated below 20kPa over a period of 64 days, whereas the control had reached 61kPa on a shorter period, demonstrating reduced TMP rise as well as lower flux decline [36,37]. Although adsorption methods have been reported to be effective, their effectiveness depends on the type of foulant. Hydrophobic foulants are effectively removed by GAC/PAC; however, they also have a low affinity for hydrophilic foulants and low-molecular-weight biopolymers, which are the main precursors of biofilm formation [38,39].

**Table S1: Summary of pre-treatment techniques in biofouling prevention**

| Pre-treatment Technique | Chemicals mainly used | Primary targets | Advantages | Limitations | Ref. |
| --- | --- | --- | --- | --- | --- |
| Adsorption | Activated carbon (granular/ powder/ biological) | Non-organic matter assimilates organic matter, metals, and microbes. | - Reversible. - Simple and can be used at a large scale. - High efficacy. | - Limited longevity. - Environmental sensitivity. - Potential toxicity. | [40–42] |
| Oxidation | Ozone, Chlorides | Molecular organics, microbial cells | - Degrades foulants. - Removes adhesions - The cake layer is loosened. | - High cost and complexity. - Limited effectiveness on certain compounds. - Potential formation of toxic by-products. | [43–45] |
| Ion Exchange | Resins | Charged NOM | - Low operational costs - Reduces organics and fouling precursors | - High capital cost - The treatment is very complex | [43,46] |
| Flocculation | PFC, PAC, PDMDAAC, FeCl_2_ | Colloids, solid particles | - Make use of eco-friendly flocculants - Reduces foulant load | - Risk of chemical residuals in the water. - Variable efficacy | [40,41,47,48] |

**Key: PFC= Perfluorocarbons PAC= Powdered activated carbon PDMDAAC= Poly (diallyl dimethylammonium chloride).**

Ion exchange (IE), on the other hand, targets charged particles like nutrients, which promotes microbial growth**.** Typically, synthetic resins that contain charged functional groups capable of exchanging ions with the feed water are utilised. By improving the elimination of organic matter naturally and removing ions like calcium that cause fouling and lower membrane permeate flux, ion exchange significantly reduces membrane biofouling [49,50]. Biologically active ion exchange (BIEX) enhanced NOM removal by approximately 50%, decreasing fouling in subsequent ultrafiltration processes [50]. After the addition of phosphorus to feedwater, the RO feedwater pretreated with ion exchange exhibited an 80% reduction in microbial growth rate compared to the control, which was not pretreated with ion exchange [51]. While IE techniques remove charged foulants, they have weak to no attraction towards uncharged and weakly charged foulants, which contribute to fouling. Some resins are also prone to fouling over time, reducing their efficiency, and may require frequent replacement.

Coagulation–flocculation is a widely used pretreatment strategy to curb membrane biofouling by aggregating NOM, biopolymers (e.g., proteins, polysaccharides), and transparent exopolymer particles (TEP) into settleable flocs, thereby reducing the concentration passing through the membrane. In a study by Li et al. (2024), seawater RO systems integrating coagulation with sand filtration managed to remove 70% of dissolved organic matter, reducing the density of the cake layer on the RO membrane [52]. The most used flocculants are chloride-based, which include polyferric chloride (PFC), polyaluminum chloride (PAC), and composite flocculants polyaluminum chloride-polydimethyldiallylammonium chloride (PAC-PDMDAAC) and PFC-PDMDAAC [47,53]. Another flocculant often used is FeCl_2;_ it has been shown to delay flux attenuation by 57% while another study indicated that utilising flocculation together with NF can increase the TOC removal from 43.6% to 60.1% [48,54]. The efficacy of several oxidation techniques, including electrochemical peroxidation, ozonation, and chlorination, as pretreatment techniques in reducing biofouling have been investigated. These methods work primarily by altering characteristics of the foulants, enhancing charge neutralisation of the membrane, or creating oxygen bubbles, thereby enhancing foulant repulsion and reducing foulant binding [44,45]. Using oxidation as a pre-treatment technique can significantly reduce the flux decline; Li et al. (2022) in their study using UF membranes demonstrated that after 3 hours of operation flux declined to 79.1% when compared to the control, which declined to 28.5% without the pre-treatment, highlighting the importance of pre-treatment [55].

## **Membrane cleaning as a biofouling mitigation strategy**

The cleaning process involves removing accumulated foulants from the membrane surface to maintain a specific permeate flux. The different cleaning methods can be classified as either physical, chemical, or biological. Physical cleaning methods use hydraulic or mechanical forces to remove foulants. In the case of chemical cleaning, the process involves adding chemicals in situ or ex situ, followed by rinsing in tanks. It has been previously reported that mature biofilms are not readily removed by chemical cleaning and to increase the chances of removing more biofilms, more chemical cycles are added [56].

### **Physical methods of biofouling mitigation**

Pre-treatment techniques usually prolong the initial attachment of the biofilm but cannot completely prevent biofouling, which will require cleaning methods to ensure the smooth, effective operation of filtration processes. Physical cleaning methods, such as backwashing and air scourging, fall under the category of cleaning biofilms once fouling has occurred. Backwashing is a technique that involves reversing the flow of water through the membrane to dislodge accumulated biofilms. Blended polyvinyl chloride/polycarbonate (PVC/PC) and nanocomposite PVC/PC/modified silver nanoparticles (PVC/PC/MAg) hollow fibre membranes were tested under continuous (**Figure S2a** and **S2c**) and intermittent filtration (**Figure S2b** and **S2d).** The effect of backwashing was pronounced on the composite membrane. The flux at the end of the continuous filtration experiments with blended and nanocomposite membranes declined by about 61% and 48%, respectively, compared to the initial flux. The average flux of the first step compared with that of the last step for the blended and nanocomposite membranes in the intermittent filtration experiments was decreased by about 16% and 7%, respectively.


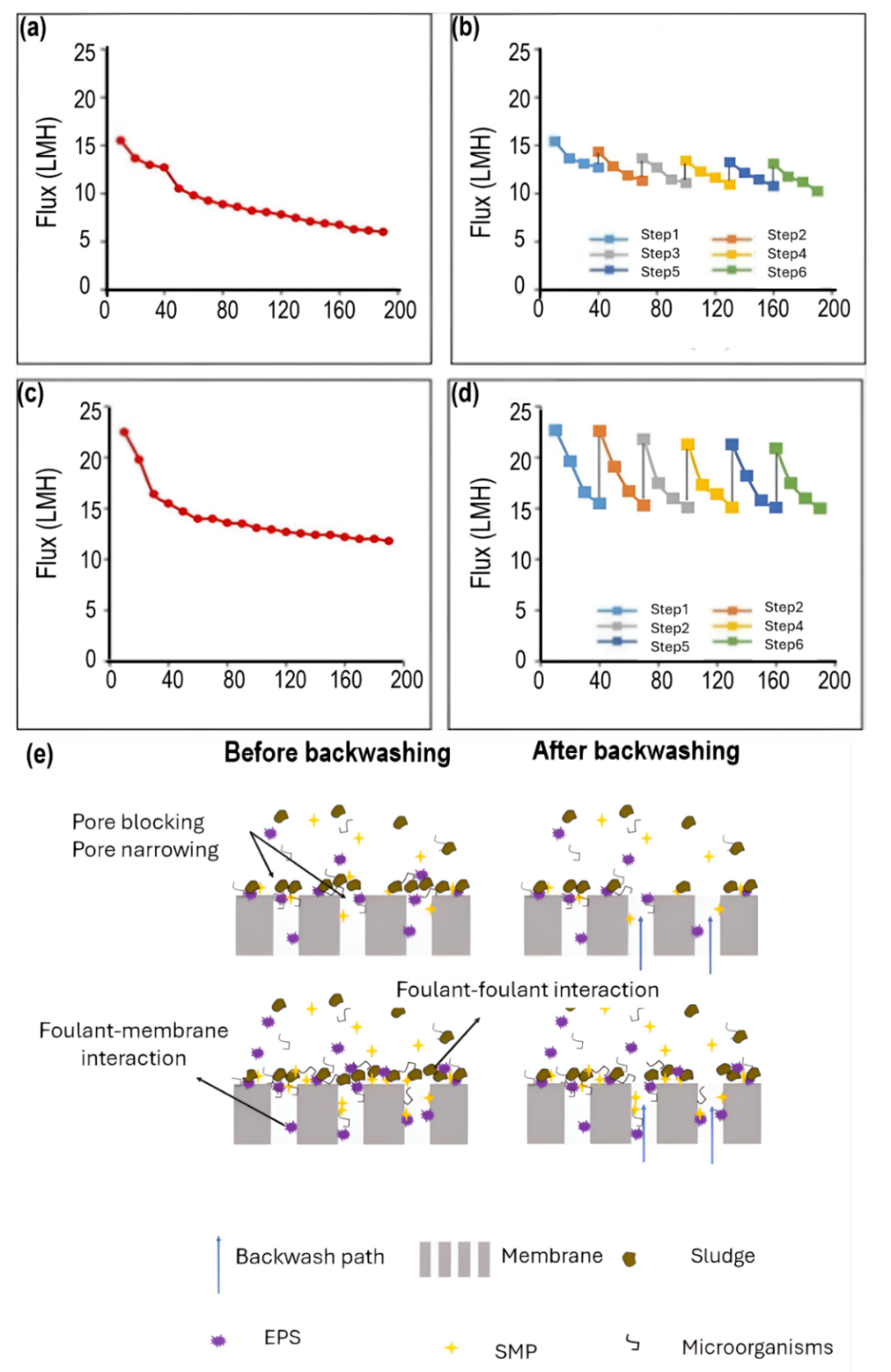


**Figure S2:** Flux vs. Time graphs of blended membrane over (a) continuous and (b) intermittent filtration experiments and nanocomposite membrane over (c) continuous and (d) intermittent filtration experiments Reproduced with permission according to the terms of the CC-BY license, Copyright 2020 [57].

In air scourging/sparging, bubbles are introduced onto the membrane surface to dislodge and disrupt the biofilm, thereby preventing its formation. It is mainly used in membrane bioreactors to induce turbulence and shear forces [58]. The efficiency of air sparging depends mainly on the size of the bubbles, operating conditions, air flow rate and membrane module configuration. It is mainly used in conjunction with other techniques such as relaxation and backwashing. When applied together with relaxation, there is less recovery of flux to 25% due to the formation of a denser biofilm, but when applied after the relaxation cycle, flux recovery was 250% [59]. When air-backwashing was employed for 10 seconds, it effectively controlled biofouling in seawater membrane distillation with just a slight decrease in flux after 4 hours of filtration at 1 bar pressure [60]. Despite the reduction in biofouling, the introduction of an air layer evidently induced crystallization on the membrane surface as evidenced by the salt crystal deposition on the membrane surface. This requires special attention when selecting suitable strategies to mitigate biofouling, as other treatment options may unintentionally accelerate the production of other types of fouling.

**1.3 Metal-based nanomaterials in membrane modifications**

Metal-based nanomaterials have gained popularity in recent years due to their excellent antimicrobial and photocatalytic characteristics, making them applicable in water purification and antifouling surfaces. Among the most widely studied are silver (Ag), titanium dioxide (TiO₂), zinc oxide (ZnO), and copper (Cu) nanoparticles [61]. Such nanomaterials exhibit antimicrobial activity through the disruption of the microbial cell membrane, interference of intracellular metabolic pathways, and generation of reactive oxygen species (ROS), predominantly under light [62,63]. Their ability to generate ROS-hydroxyl radicals, superoxide anions, and hydrogen peroxide produces the oxidative stress that leads to damage in the DNA of foulants, proteins, and lipids of microorganisms, which ultimately leads to cell death [64]. Silver nanoparticles (AgNPs) possess antimicrobial properties against bacteria, fungi, and viruses, in which they use the mechanisms illustrated in **Figure S3a** to disrupt and destroy the biofilm [65]. With their flexibility in being embedded within filtration membranes, polymeric matrices, and surface coatings, they effectively inhibit microbial adhesion and biofilm formation [62]. AgNPs coatings were used to modify hollow fibre MF membranes and tested for their antibiofouling capabilities in an MBR with high mixed liquor suspended solids (MLSS) for 60 days. The modified membrane exhibited better antifouling behaviour with a 59.7% decrease in specific flux as compared to the unmodified membrane, which had an 81.8% decrease [66]. Furthermore, silver can be grafted into nanopollens as indicated by Wang et al. (2022), who introduced silver-immobilized silica nanopollens (onto PVDF membranes. They were tested over 75 days, and the modified membranes displayed excellent antifouling properties with a filtration cycle of 32 days, while the unmodified membranes had a cycle of 16 days. The microbial cells formed a fouling layer most of them aggregated into clusters of live cells, while organic foulants also formed a thicker fouling layer on the unmodified membrane surfaces. In contrast, many dead cells were observed on the Ag@Snp-modified membranes and a thinner layer of organic foulants was also observed, showing the ability of the AgNPs in mitigating biofouling [67]. Sun et al. (2024) investigated the improvement of RO membranes through the incorporation of AgNPs using the tannic acid (TA) and diethylenetriamine (DETA) co-deposition method. AgNPs exhibited enhanced antibiofouling activity, achieving bacterial reduction rates of 98.23% for *Escherichia coli* and 99.83% for *Staphylococcus aureus* [68]. Cobalt, in the form of nanoengineered CoFe_2_O_4_ membranes_,_ has also been applied in salts and Naproxen removal from water solution and evaluated for antimicrobial removal. The antimicrobial effect was evidenced by the SEM micrographs in **FigureS3b-S3e** which shows that before treatment, the *S. aureus* cells were spherical, intact, and well-spaced **(Figure S3b)** but after 180 min exposure to CoFe₂O₄ membranes **(Figure S3c- S3e)**, the cells became shriveled, wrinkled, and aggregated, indicating cytosolic leakage and loss of cellular function[69].

**
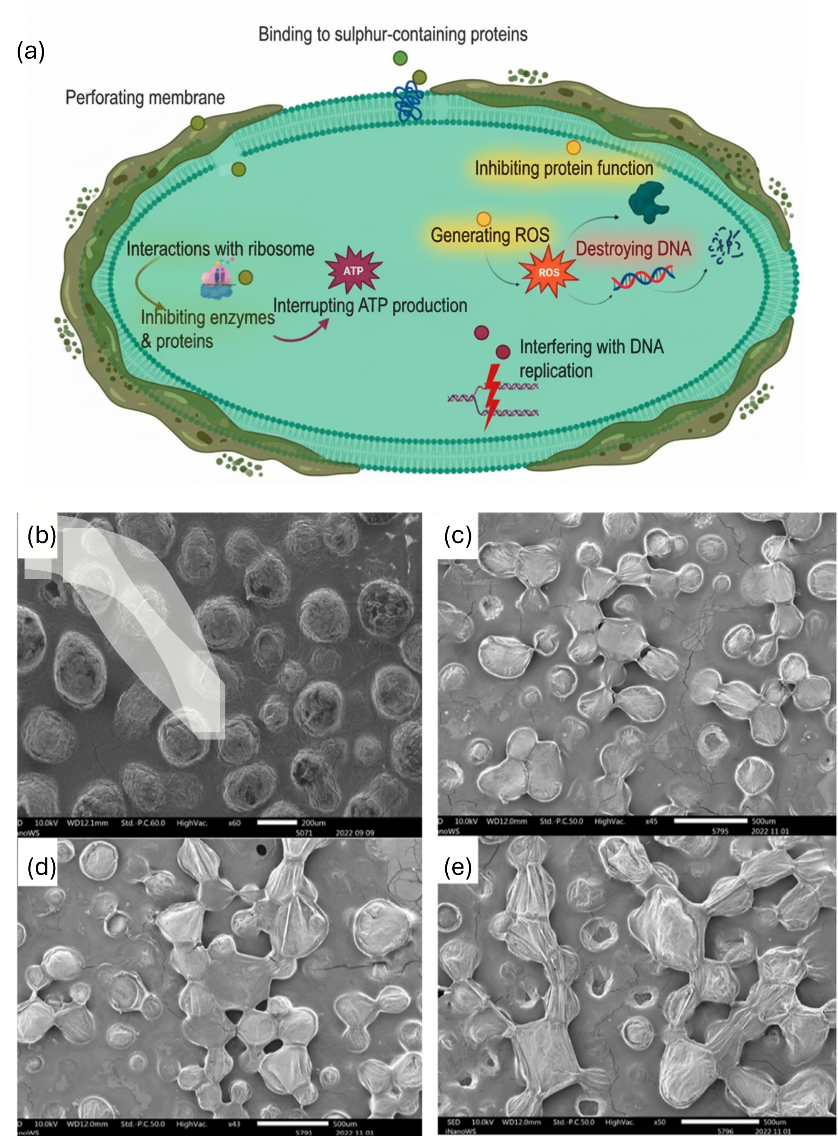
**

**Figure S3:** (a) The general antifouling actions of AgNPs. Reproduced with permission according to the terms of the CC-BY license, The authors published by Springer nature [65], SEM micrographs of S. aureus treated under light (b) untreated cells (control), (c-e) treated cells Reproduced with permission according to the terms of the CC-BY license, Copyright 2023 [69]

**Table S2: Comparative study of carbon-based nanomaterials used in membrane modifications**

| Carbon-based nanomaterials | Membrane material | Study highlights | Ref. |
| --- | --- | --- | --- |
| Graphene oxide (GO) | 0.5wt % Sulfonated PVDF membranes doped with GO | - There was enhanced hydrophilicity - Improved antimicrobial activity as compared to the control - Improved water flux of 309 L/m²·h·bar compared to 112 L/m²·h·bar of the pure PVDF membrane - Improved antifouling behavior evidenced by Bovine Serum albumin (BSA) >55% | [70] |
|  | PVDF membranes modified with GO–AgNPs | - The modified membrane demonstrated improved hydrophilicity and mechanical strength - Inhibited the growth of *E. coli* | [71] |
|  | PVDF membrane coated with a GO-chitosan layer | - Increase in dye rejection up 96% - Greater flux recovery compared to the control - Lower irreversible fouling by bsa - Increased hydrophilicity - Reduced pore blockage | [72] |
|  | Thin‑film composite membranes modified with GO | - Reactive oxygen species (ROS) generation by GO functional groups - Up to ~69% reduction in *E. Coli* CFUs; 58–77% reduction in cell adhesion | [73] |
| Reduced Graphene oxide (rGO) | rGO layer with CNT protective layer on PAN support | - Enhanced hydrophilicity - 50% less fouling - Enhanced foulant repulsion - 96% after back flushing | [74] |
|  | rGO incorporated in bacterial nanocellulose UF membrane | - Under illumination, flux declines at a slower rate than the unmodified - There was photothermal inactivation of bacteria, leading to great antifouling | [75] |
|  | PES blended with ZnO-rGO nanocomposite | - 60–80% bacterial viability reduction (with ZnO or light) - There was improved hydrophilicity as compared to the unblended | [76] |
| GQDs | Thin‑film composite FO membrane modified with ~100 ppm amino‑GQD | - There was an increase in permeability from 16.94 to 58.32 L/m²·h - A 96% flux recovery after cleaning(vs 63% for unmodified) - Improved hydrophilicity, - Reduced bacterial adhesion | [77] |
|  | Thin-film nanocomposite NF with GQDs in tannic acid layer | - Pure water flux rose to 23.33 L/m²·h (1.5× compared to pristine TA‑TFC) - Improved antifouling performance due to the smoothening of the membrane surface as well as increased hydrophilicity | [78] |
|  | Cellulose acetate membranes prepared via phase inversion with GQDs | - Up to 85–90% protein rejection - Achieved 70–80% bacterial reduction - Flux was improved and there was 99% salt rejection as well | [79] |
| CNTs | PVC UF membrane, with low-voltage cathodic assistance blended with CNT | - High water permeability - High flux 196 L/m²·h at 0.2 mpa under conductive mode - Reduced humics adhesion on the membrane - Stable flux during electro-assisted filtration | [80] |
|  | Microwave-assisted in‑situ growth of CNTs on NF270 (polyamide) membrane | - Maintained constant water permeability and flux - 14% improvement on ion rejection, no flux loss - Improved antifouling performance | [81] |
|  | Vertically aligned CNT membranes with nanochannels | - Achieved a flux of 66-270 LMH, which is 2.4-9.8 times higher than the commercial RO membranes - There was improved antifouling performance, inhibiting the formation of biofilms - There was improved antifouling performance | [82] |
|  | PES UF membranes blended with ZnO and MWCNT nanofillers | - Reached permeate flux of 40 L/m²·h (significantly higher than the unblended PES) - Lower relative flux reduction (RFR) and high flux recovery ratio (FRR) - Comparatively less bacterial adhesion | [83] |

**Table S3: Enzymes used in biofouling mitigation applications**

| Enzyme | Type of foulants | Action of the enzymes | Type of membrane  used | Application highlights | Ref. |
| --- | --- | --- | --- | --- | --- |
| Proteinase-k  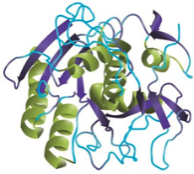 | *S. aureus*  *E. coli* | Degrade proteins | Polystyrene | - Achieved > 91% reduction in biofilm and inhibited further biofilm formation | [84] |
| Trypsin- EDTA  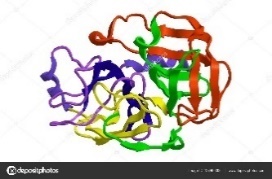 | Protein-based foulants | Breakdown proteins into peptides | RO | - Reduced the proteins’ ability to adhere to the membrane - Performed better than proteinase K alone, leading to a 71% reduction in biomass when used together with other enzymes | [85] |
| Papain  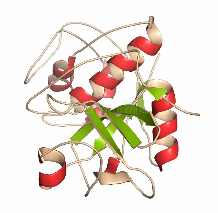 | c and Listeria monocytogenes | Degraded the biofilm matrix | RO | - led to 56.2% and 59% biofilm inhibition and 54.9% and 55.6% biofilm eradication at 50 mg/L and 100mg/L, respectively | [86] |
| α-amylase  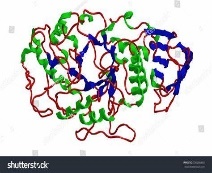 | Polysaccharide-based biofilms | Hydrolysis of starch and glycogen into simpler sugars | RO | - Reduction of the structural integrity of the biofilm matrix - 92-97% removal of preformed biofilm | [87] |
| Lysozyme  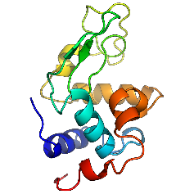 | Bacterial peptidoglycan, Staphylococcus aureus | Hydrolyses glycosidic bonds in peptidoglycan | Polystrene surfaces | - Showed over 80% reduction in bacterial colony-forming units on the membrane surface within 24 hours of contact (12.5% viability for *E. coli* and 8.3% for *S. aureus*) | [88] |
| β-Mannosidase  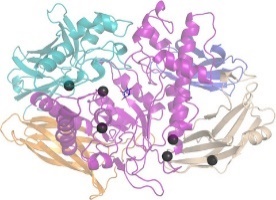 | Polysaccharides | Hydrolyses mannosidic bonds | RO | - Breaks down the structural components of biofilms, - it achieved a 0.284 log reduction in biofilm formation, outperforming other individual enzymes like Trypsin-EDTA, Proteinase K, α-Amylase, and Alginate Lyase under similar conditions. | [85] |
| Endoglucanase  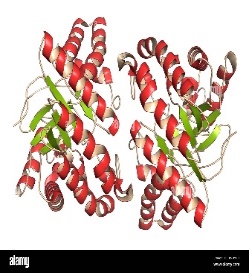 | Cellulose | Hydrolyses internal glycosidic bonds into simpler oligosaccharides | RO | - Demonstrated 76.8% reduction of *S aureus* and 61.7% *P. aeruginosa* | [89] |
| Alginate lyase  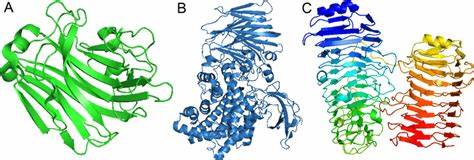 | Alginate | Cleaves the alginate polymer into smaller oligosaccharides | RO | - -Disrupts the biofilm matrix, which is essential for the structural integrity of the biofilm - retained 80% of its original activity after 21 days | [90] |
|  | Alginate  Pseudomonas aeruginosa | Break down the polysaccharides | UF/MF | - Inhibit the growth of the biofilm - Foulant resistance dropped by 82% compared to the control | [91] |
| Acylase  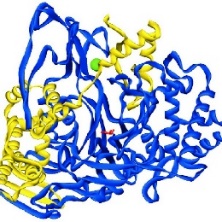 | Pseudomonas aeruginosa | Disrupt quorum sensing among the microorganisms | RO  Polystrene membrane | - Reduced the production of EPS - Altered the biofilm matrix - reduced biofilm formation by 20% for *A. hydrophila* and 24% for *P. putida* on RO membranes, *and* 60% and 73%, respectively, on polystyrene membranes | [92] |

**References**

[1] Costa FCR, Ricci BC, Teodoro B, Koch K, Drewes JE, Amaral MCS. Biofouling in membrane distillation applications - a review. Desalination 2021;516. https://doi.org/10.1016/j.desal.2021.115241.

[2] van der Marel P, Zwijnenburg A, Kemperman A, Wessling M, Temmink H, van der Meer W. Influence of membrane properties on fouling in submerged membrane bioreactors. J Memb Sci 2010;348:66–74. https://doi.org/10.1016/j.memsci.2009.10.054.

[3] Melo LF, Bott TR. Biofouling in water systems. Exp Therm Fluid Sci 1997;14:375–81. https://doi.org/10.1016/S0894-1777(96)00139-2.

[4] O’Toole G, Kaplan HB, Kolter R. Biofilm Formation as Microbial Development. Annu Rev Microbiol 2000;54:49–79. https://doi.org/10.1146/annurev.micro.54.1.49.

[5] Binahmed S, Hasane A, Wang Z, Mansurov A, Romero-Vargas Castrillón S. Bacterial Adhesion to Ultrafiltration Membranes: Role of Hydrophilicity, Natural Organic Matter, and Cell-Surface Macromolecules. Environ Sci Technol 2018;52:162–72. https://doi.org/10.1021/acs.est.7b03682.

[6] Suwarno SR, Hanada S, Chong TH, Goto S, Henmi M, Fane AG. The effect of different surface conditioning layers on bacterial adhesion on reverse osmosis membranes. Desalination 2016;387:1–13. https://doi.org/10.1016/j.desal.2016.02.029.

[7] Hakami MW, Alkhudhiri A, Al-Batty S, Zacharof MP, Maddy J, Hilal N. Ceramic microfiltration membranes in wastewater treatment: Filtration behavior, fouling and prevention. Membranes (Basel) 2020;10:1–34. https://doi.org/10.3390/membranes10090248.

[8] Asif MB, Zhang Z. Ceramic membrane technology for water and wastewater treatment: A critical review of performance, full-scale applications, membrane fouling and prospects. Chemical Engineering Journal 2021;418. https://doi.org/10.1016/j.cej.2021.129481.

[9] Tafa A, Bernstein A, Elsner M, Bakkour R. Role of membrane porosity in passive sampling of aquatic contaminants for stable isotope analysis: enhancement of analyte accumulation rates and selectivity. Anal Bioanal Chem 2025. https://doi.org/10.1007/s00216-025-05756-9.

[10] Khan B, Haider S, Khurram R, Wang Z, Wang X. Preparation of an ultrafiltration (UF) membrane with narrow and uniform pore size distribution via etching of SiO2 nano-particles in a membrane matrix. Membranes (Basel) 2020;10:1–18. https://doi.org/10.3390/membranes10070150.

[11] Zhang H, Zhu S, Yang J, Ma A. Advancing Strategies of Biofouling Control in Water-Treated Polymeric Membranes. Polymers (Basel) 2022;14. https://doi.org/10.3390/polym14061167.

[12] Ismail AF, Matsuura T. Membrane module. Membrane Separation Processes, Elsevier; 2022, p. 165–92. https://doi.org/10.1016/B978-0-12-819626-7.00009-0.

[13] Siddiqui A. Biofouling Control in Spiral-Wound Membrane Systems: Impact of Feed Spacer Modification and Biocides Dissertation by. n.d.

[14] Picioreanu C, Vrouwenvelder JS, van Loosdrecht MCM. Three-dimensional modeling of biofouling and fluid dynamics in feed spacer channels of membrane devices. J Memb Sci 2009;345:340–54. https://doi.org/10.1016/j.memsci.2009.09.024.

[15] Farhat NM, Vrouwenvelder JS, Van Loosdrecht MCM, Bucs SS, Staal M. Effect of water temperature on biofouling development in reverse osmosis membrane systems. Water Res 2016;103:149–59. https://doi.org/10.1016/j.watres.2016.07.015.

[16] López D, Vlamakis H, Kolter R. Biofilms. Cold Spring Harb Perspect Biol 2010;2. https://doi.org/10.1101/cshperspect.a000398.

[17] Pichardo-Romero D, Garcia-Arce ZP, Zavala-Ramírez A, Castro-Muñoz R. Current advances in biofouling mitigation in membranes for water treatment: An overview. Processes 2020;8. https://doi.org/10.3390/pr8020182.

[18] Uçar A, González-Fernández E, Staderini M, Murray AF, Mount AR, Bradley M. pH-Activated Dissolvable Polymeric Coatings to Reduce Biofouling on Electrochemical Sensors. J Funct Biomater 2023;14. https://doi.org/10.3390/jfb14060329.

[19] Radu AI, Vrouwenvelder JS, van Loosdrecht MCM, Picioreanu C. Effect of flow velocity, substrate concentration and hydraulic cleaning on biofouling of reverse osmosis feed channels. Chemical Engineering Journal 2012;188:30–9. https://doi.org/10.1016/j.cej.2012.01.133.

[20] Chuanwen S, Haiqiao W, Qi Y, Shiqiang C, Xun L, Hanyang W. Experimental study of the flux Law of flat ceramic membranes under different pressures. Water Pract Technol 2020;15:416–25. https://doi.org/10.2166/wpt.2020.028.

[21] Bogler A, Lin S, Bar-Zeev E. Biofouling of membrane distillation, forward osmosis and pressure retarded osmosis: Principles, impacts and future directions. J Memb Sci 2017;542:378–98. https://doi.org/10.1016/j.memsci.2017.08.001.

[22] Leong CL, Bilad MR, Shamsuddin N, Suhaimi H, Arahman N, Giwa A, Yusuf A. Ultralow pressure membrane filtration for water and wastewater treatment. Current Developments in Biotechnology and Bioengineering, Elsevier; 2023, p. 113–41. https://doi.org/10.1016/B978-0-443-19180-0.00020-1.

[23] Abdelrasoul A, Doan H, Lohi A. Fouling in Membrane Filtration and Remediation Methods. Mass Transfer - Advances in Sustainable Energy and Environment Oriented Numerical Modeling, InTech; 2013, p. 195–218. https://doi.org/10.5772/52370.

[24] Zhang Z, Li X, Liu H, Zhou T, Wang Z, Nghiem LD, Wang Q. Biofouling control of reverse osmosis membrane using free ammonia as a cleaning agent. J Memb Sci 2024;694. https://doi.org/10.1016/j.memsci.2024.122414.

[25] Huang S, Voutchkov N, Jiang S. Balancing carbon, nitrogen and phosphorus concentration in seawater as a strategy to prevent accelerated membrane biofouling. Water Res 2019;165. https://doi.org/10.1016/j.watres.2019.114978.

[26] Vanysacker L, Declerck P, Bilad MR, Vankelecom IFJ. Biofouling on microfiltration membranes in MBRs: Role of membrane type and microbial community. J Memb Sci 2014;453:394–401. https://doi.org/10.1016/j.memsci.2013.11.024.

[27] Kilmer NT, Huss RL, George CC, Stennett EMS. The influence of ion identity and ionic strength on membrane biofouling of a binary protein solution. Sep Purif Technol 2021;255. https://doi.org/10.1016/j.seppur.2020.117769.

[28] Bouayed N, Cavalier A, Lafforgue C, Dietrich N, Lee C-H, Guigui C. Hydrodynamics Characterization of the Impact of Free-Moving Particles in an Air-Lift Membrane Bioreactor. Ind Eng Chem Res 2020;59:7943–54. https://doi.org/10.1021/acs.iecr.9b06749.

[29] Bucs SS, Farhat N, Siddiqui A, Valladares Linares R, Radu A, Kruithof JC, Vrouwenvelder JS. Development of a setup to enable stable and accurate flow conditions for membrane biofouling studies. Desalination Water Treat 2016;57:12893–901. https://doi.org/10.1080/19443994.2015.1057037.

[30] Qamar A, Kerdi S, Ali SM, Shon HK, Vrouwenvelder JS, Ghaffour N. Novel hole-pillar spacer design for improved hydrodynamics and biofouling mitigation in membrane filtration. Sci Rep 2021;11. https://doi.org/10.1038/s41598-021-86459-w.

[31] Pan M, Li H, Han X, Jiang S, Diao Y, Ma W, Li X, Qin J, Yao J, Wang Z. Impact of Hydrodynamic Conditions on the Production and Distribution of Extracellular Polymeric Substance in River Biofilms. Water (Switzerland) 2023;15. https://doi.org/10.3390/w15213821.

[32] Du X, Wang Y, Leslie G, Li G, Liang H. Shear stress in a pressure-driven membrane system and its impact on membrane fouling from a hydrodynamic condition perspective: a review. Journal of Chemical Technology and Biotechnology 2017;92:463–78. https://doi.org/10.1002/jctb.5154.

[33] Chun ALM, Mosayyebi A, Butt A, Carugo D, Salta M. Early biofilm and streamer formation is mediated by wall shear stress and surface wettability: A multifactorial microfluidic study. Microbiologyopen 2022;11. https://doi.org/10.1002/mbo3.1310.

[34] Fanesi A, Lavayssière M, Breton C, Bernard O, Briandet R, Lopes F. Shear stress affects the architecture and cohesion of Chlorella vulgaris biofilms. Sci Rep 2021;11. https://doi.org/10.1038/s41598-021-83523-3.

[35] Zhang B, Mao X, Shen Y, Ma T, Liu B, Shi W. Enhanced performance and mechanism of adsorption pretreatment for alleviating membrane fouling in AGMBR: Impact of structural variations in carbon adsorbents. Science of the Total Environment 2024;940. https://doi.org/10.1016/j.scitotenv.2024.173702.

[36] Morales N, Mery-Araya C, Guerra P, Poblete R, Chacana-Olivares J. Mitigation of Membrane Fouling in Membrane Bioreactors Using Granular and Powdered Activated Carbon: An Experimental Study. Water (Basel) 2024;16:2556. https://doi.org/10.3390/w16172556.

[37] Sun W, Liu J, Chu H, Dong B. Pretreatment and membrane hydrophilic modification to reduce membrane fouling. Membranes (Basel) 2013;3:226–41. https://doi.org/10.3390/membranes3030226.

[38] Tang X, Pronk W, Traber J, Liang H, Li G, Morgenroth E. Integrating granular activated carbon (GAC) to gravity-driven membrane (GDM) to improve its flux stabilization: Respective roles of adsorption and biodegradation by GAC. Science of the Total Environment 2021;768. https://doi.org/10.1016/j.scitotenv.2020.144758.

[39] Gur-Reznik S, Katz I, Dosoretz CG. Removal of dissolved organic matter by granular-activated carbon adsorption as a pretreatment to reverse osmosis of membrane bioreactor effluents. Water Res 2008;42:1595–605. https://doi.org/10.1016/j.watres.2007.10.004.

[40] Zhang J, Li G, Yuan X, Li P, Yu Y, Yang W, Zhao S. Reduction of Ultrafiltration Membrane Fouling by the Pretreatment Removal of Emerging Pollutants: A Review. Membranes (Basel) 2023;13. https://doi.org/10.3390/membranes13010077.

[41] Matebese F, Moutloali RM. Integrating Ultrafiltration Membranes with Flocculation and Activated Carbon Pretreatment Processes for Membrane Fouling Mitigation and Metal Ion Removal from Wastewater. ACS Omega 2023;8:9074–85. https://doi.org/10.1021/acsomega.2c03524.

[42] Nguyen T, Roddick FA, Fan L. Biofouling of water treatment membranes: A review of the underlying causes, monitoring techniques and control measures. Membranes (Basel) 2012;2:804–40. https://doi.org/10.3390/membranes2040804.

[43] Sun W, Liu J, Chu H, Dong B. Pretreatment and membrane hydrophilic modification to reduce membrane fouling. Membranes (Basel) 2013;3:226–41. https://doi.org/10.3390/membranes3030226.

[44] Xu S, Li G, Zhou S, Shi Z, Liu B. Effect of Electrochemical Pre-Oxidation for Mitigating Ultrafiltration Membrane Fouling Caused by Extracellular Organic Matter. Water (Switzerland) 2023;15. https://doi.org/10.3390/w15122235.

[45] Zeng W, Zhang H, Zhao J, Wang J, Bai L, Li G, Liang H. Synergistic roles of oxidation and self-aggregation in efficient ultrafiltration membrane fouling alleviation using a flow-through Sb-SnO2 anode during wastewater reclamation. Water Res 2024;249. https://doi.org/10.1016/j.watres.2023.121003.

[46] Kato S, Kansha Y. Comprehensive review of industrial wastewater treatment techniques. Environmental Science and Pollution Research 2024;31:51064–97. https://doi.org/10.1007/s11356-024-34584-0.

[47] Zhang S, Zheng H, Tang X, Zhao C, Zheng C, Gao B. Sterilization by flocculants in drinking water treatment. Chemical Engineering Journal 2020;382. https://doi.org/10.1016/j.cej.2019.122961.

[48] Yadai T, Suzuki Y. Development of softening and ballasted flocculation as a pretreatment process for seawater desalination through a reverse osmosis membrane. NPJ Clean Water 2023;6. https://doi.org/10.1038/s41545-023-00226-0.

[49] Dong H, Lin T, SenGupta AK. Field validation of multifunctional ion exchange process for reverse osmosis pretreatment and phosphate recovery during impaired water reuse. Journal of Water Process Engineering 2020;36. https://doi.org/10.1016/j.jwpe.2020.101347.

[50] Schulz M, Winter J, Wray H, Barbeau B, Bérubé P. Biologically active ion exchange (BIEX) for NOM removal and membrane fouling prevention. Water Sci Technol Water Supply 2017;17:1178–84. https://doi.org/10.2166/ws.2017.016.

[51] Gkoutzamani I. Ion Exchange as Pretreatment of Municipal Wastewater Effluent for Reverse Osmosis Desalination Assessment of treatment performance and prediction of fouling potential on downstream reverse osmosis. n.d.

[52] Li Q, Xie L, Xu S, Zhang W. Comparison of Coagulation-Integrated Sand Filtration and Ultrafiltration for Seawater Reverse Osmosis Pretreatment. Membranes (Basel) 2024;14. https://doi.org/10.3390/membranes14060125.

[53] Shen X, Gao B, Guo K, Yue Q. Application of composite flocculants for removing organic matter and mitigating ultrafiltration membrane fouling in surface water treatment: The role of composite ratio. Environ Sci (Camb) 2019;5:2242–50. https://doi.org/10.1039/c9ew00528e.

[54] Zhao J, Jiang T, Cheng Y, Cao J. The influence of micro-flocculation on membrane fouling during ultrafiltration of dissolved organic matter. Journal of Water Process Engineering 2023;56. https://doi.org/10.1016/j.jwpe.2023.104361.

[55] Li Y, Wang Y, Liao M, Su F, Zhang Y, Peng L. Effects of electroflocculation/oxidation pretreatment on the fouling characteristics of ultrafiltration membranes. Water Science and Technology 2022;85:1079–89. https://doi.org/10.2166/wst.2022.016.

[56] AlSawaftah N, Abuwatfa W, Darwish N, Husseini GA. A Review on Membrane Biofouling: Prediction, Characterization, and Mitigation. Membranes (Basel) 2022;12. https://doi.org/10.3390/membranes12121271.

[57] Chapalaghi M, Ahsani M, Ghofrani B, Ranjbaran N, Yegani R. A step-by-step assessment of the backwashing process impact on the fouling mitigation of blended PVC/PC and nanocomposite PVC/PC/MAg membranes in a membrane bioreactor (MBR) treating pharmaceutical wastewater. Chemical Engineering Research and Design 2022;188:831–45. https://doi.org/10.1016/j.cherd.2022.10.020.

[58] Kim Y, Li S, Ghaffour N. Evaluation of different cleaning strategies for different types of forward osmosis membrane fouling and scaling. J Memb Sci 2020;596. https://doi.org/10.1016/j.memsci.2019.117731.

[59] Fortunato L, Ranieri L, Naddeo V, Leiknes TO. Fouling control in a gravity-driven membrane (GDM) bioreactor treating primary wastewater by using relaxation and/or air scouring. J Memb Sci 2020;610. https://doi.org/10.1016/j.memsci.2020.118261.

[60] Harimawan A, Wonoputri V, Ariel J, Michell, Julian H. Biofouling control of membrane distillation for seawater desalination: Effect of air-backwash and chemical cleaning on biofouling formation. Biofouling 2022;38:889–902. https://doi.org/10.1080/08927014.2022.2146496.

[61] Blanco-Covián L, Campello-García JR, Blanco-López MC, Miranda-Martínez M. Synthesis, characterization and evaluation of the antibiofouling potential of some metal and metal oxide nanoparticles. Applied Sciences (Switzerland) 2020;10. https://doi.org/10.3390/app10175864.

[62] Ren S, Guo N, Li J, Wang Y. Integration of antibacterial and photocatalysis onto polyethersulfone membrane for fouling mitigation and contaminant degradation. J Environ Chem Eng 2023;11. https://doi.org/10.1016/j.jece.2023.110401.

[63] Nazerah A, Ismail AF, Jaafar J. Incorporation of bactericidal nanomaterials in development of antibacterial membrane for biofouling mitigation: A mini review. J Teknol 2016;78:2180–3722. https://doi.org/10.11113/jt.v78.10067.

[64] Fallah S, Yusefi-Tanha E, Peralta-Videa JR. Interaction of nanoparticles and reactive oxygen species and their impact on macromolecules and plant production. Plant Nano Biology 2024;10. https://doi.org/10.1016/j.plana.2024.100105.

[65] Afkhami F, Forghan P, Gutmann JL, Kishen A. Silver Nanoparticles and Their Therapeutic Applications in Endodontics: A Narrative Review. Pharmaceutics 2023;15:715. https://doi.org/10.3390/pharmaceutics15030715.

[66] Le HQ, Sowe A, Chen SS, Duong CC, Ray SS, Cao TND, Nguyen NC. Exploring nanosilver-coated hollow fiber microfiltration to mitigate biofouling for high loading membrane bioreactor. Molecules 2019;24. https://doi.org/10.3390/molecules24122345.

[67] Wang X, Guo Y, Wang T, Zhang X. Silver@silica nanopollen modified membranes for wastewater treatment in membrane bioreactors: limited adverse effects on microorganisms and compelling antifouling properties. Environ Sci (Camb) 2022;8:640–7. https://doi.org/10.1039/D1EW00881A.

[68] Sun Y, Yong ZX, Xie X, Ma X, Xu C, Hu B, He JJ, Guo Y, Bai B. Improving antifouling performance of FO membrane by surface immobilization of silver nanoparticles based on a tannic acid: diethylenetriamine precursor layer for municipal wastewater treatment. Environmental Science and Pollution Research 2024;31:30988–1000. https://doi.org/10.1007/s11356-024-33312-y.

[69] Chakachaka VM, Mahlangu OT, Tshangana CS, Mamba BB, Muleja AA. Highly adhesive CoFe2O4 nanoengineered PES membranes for salts and Naproxen removal and antimicrobial activities. J Memb Sci 2023;676. https://doi.org/10.1016/j.memsci.2023.121612.

[70] Zahid M, Khalid T, Rehan ZA, Javed T, Akram S, Rashid A, Mustafa SK, Shabbir R, Mora-Poblete F, Asad MS, Liaquat R, Hassan MM, Amin MA, Shakoor HA. Fabrication and characterization of sulfonated graphene oxide (Sgo) doped pvdf nanocomposite membranes with improved anti-biofouling performance. Membranes (Basel) 2021;11. https://doi.org/10.3390/membranes11100749.

[71] Li J, Liu X, Lu J, Wang Y, Li G, Zhao F. Anti-bacterial properties of ultrafiltration membrane modified by graphene oxide with nano-silver particles. J Colloid Interface Sci 2016;484:107–15. https://doi.org/10.1016/j.jcis.2016.08.063.

[72] Ndeh NT, Sairiam S, Nuisin R. Graphene oxide-chitosan coated PVDF adsorptive microfiltration membrane: Enhancing dye removal and antifouling properties. Int J Biol Macromol 2024;282. https://doi.org/10.1016/j.ijbiomac.2024.137005.

[73] Rodríguez BE, Armendariz-Ontiveros MM, Quezada R, Huitrón-Segovia EA, Estay H, García AG, García A. Influence of multidimensional graphene oxide (Go) sheets on anti-biofouling and desalination performance of thin-film composite membranes: Effects of go lateral sizes and oxidation degree. Polymers (Basel) 2020;12:1–24. https://doi.org/10.3390/polym12122860.

[74] Wei G, Dong J, Bai J, Zhao Y, Li Y. Structurally Stable, Antifouling, and Easily Renewable Reduced Graphene Oxide Membrane with a Carbon Nanotube Protective Layer. Environ Sci Technol 2019;53:11896–903. https://doi.org/10.1021/acs.est.9b03129.

[75] Jiang Q, Ghim D, Cao S, Tadepalli S, Liu KK, Kwon H, Luan J, Min Y, Jun YS, Singamaneni S. Photothermally Active Reduced Graphene Oxide/Bacterial Nanocellulose Composites as Biofouling-Resistant Ultrafiltration Membranes. Environ Sci Technol 2019;53:412–21. https://doi.org/10.1021/acs.est.8b02772.

[76] Zhang W, Huang H, Bernstein R. Zwitterionic hydrogel modified reduced graphene oxide/ZnO nanocomposite blended membrane with high antifouling and antibiofouling performances. J Colloid Interface Sci 2022;613:426–34. https://doi.org/10.1016/j.jcis.2021.12.194.

[77] Li N, Zhang Y, Li P, Zhu B, Wang W, Xu Z. Enhanced permeability and biofouling mitigation of forward osmosis membranes via grafting graphene quantum dots. Front Chem Sci Eng 2023;17:1470–83. https://doi.org/10.1007/s11705-023-2329-5.

[78] Zhang C, Wei K, Zhang W, Bai Y, Sun Y, Gu J. Graphene Oxide Quantum Dots Incorporated into a Thin Film Nanocomposite Membrane with High Flux and Antifouling Properties for Low-Pressure Nanofiltration. ACS Appl Mater Interfaces 2017;9:11082–94. https://doi.org/10.1021/acsami.6b12826.

[79] Fadl EA, Elsahy O, Kandil S, Morsy A. Enhancing the performance and antifouling properties of reverse osmosis membranes by graphene quantum dots for water desalination. Appl Water Sci 2025;15. https://doi.org/10.1007/s13201-025-02410-8.

[80] Chen X, Gao J, Song Y, Gong Y, Qi M, Hao R. Fabrication of a high water flux conductive mwcnts/pvc composite membrane with effective electrically enhanced antifouling behavior. Coatings 2021;11. https://doi.org/10.3390/coatings11121548.

[81] Vargas-Figueroa C, Pino-Soto L, Beratto-Ramos A, Tapiero Y, Rivas BL, Berrio ME, Melendrez MF, Bórquez RM. In-Situ Modification of Nanofiltration Membranes Using Carbon Nanotubes for Water Treatment. Membranes (Basel) 2023;13. https://doi.org/10.3390/membranes13070616.

[82] Baek Y, Kim C, Seo DK, Kim T, Lee JS, Kim YH, Ahn KH, Bae SS, Lee SC, Lim J, Lee K, Yoon J. High performance and antifouling vertically aligned carbon nanotube membrane for water purification. J Memb Sci 2014;460:171–7. https://doi.org/10.1016/j.memsci.2014.02.042.

[83] Snigdha S, Thomas S, Radhakrishnan EK, Kalarikkal N. Engineered Antimicrobial Surfaces. Singapore: Springer Singapore; 2020. https://doi.org/10.1007/978-981-15-4630-3.

[84] Shukla SK, Subba Rao T. Staphylococcus aureus biofilm removal by targeting biofilm-associated extracellular proteins. Indian Journal of Medical Research, Supplement 2017;146:1–8. https://doi.org/10.4103/ijmr.IJMR_410_15.

[85] Khani M, Hansen MF, Knøchel S, Rasekh B, Ghasemipanah K, Zamir SM, Nosrati M, Burmølle M. Antifouling potential of enzymes applied to reverse osmosis membranes. Biofilm 2023;5. https://doi.org/10.1016/j.bioflm.2023.100119.

[86] Mohamed SH, Mohamed MSM, Khalil MS, Mohamed WS, Mabrouk MI. Antibiofilm activity of papain enzyme against pathogenic Klebsiella pneumoniae. J Appl Pharm Sci 2018;8:163–8. https://doi.org/10.7324/JAPS.2018.8621.

[87] Lahiri D, Nag M, Banerjee R, Mukherjee D, Garai S, Sarkar T, Dey A, Sheikh HI, Pathak SK, Edinur HA, Pati S, Ray RR. Amylases: Biofilm Inducer or Biofilm Inhibitor? Front Cell Infect Microbiol 2021;11. https://doi.org/10.3389/fcimb.2021.660048.

[88] Li S, Zhao S, Pei J, Wang H, Meng H, Vrouwenvelder JS, Li Z. Stimuli-Responsive Lysozyme Nanocapsule Engineered Microfiltration Membranes with a Dual-Function of Anti-Adhesion and Antibacteria for Biofouling Mitigation. ACS Appl Mater Interfaces 2021;13:32205–16. https://doi.org/10.1021/acsami.1c07445.

[89] Karmakar M, Lahiri D, Nag M, Dutta B, Dash S, Sarkar T, Pandit S, Upadhye VJ, Ray RR. Purification, Characterization, and Application of Endoglucanase from Rhizopus oryzae as Antibiofilm Agent. Appl Biochem Biotechnol 2023;195:5439–57. https://doi.org/10.1007/s12010-022-04043-y.

[90] Meshram P, Dave R, Joshi H, Dharani G, Kirubagaran R, Venugopalan VP. A fence that eats the weed: Alginate lyase immobilization on ultrafiltration membrane for fouling mitigation and flux recovery. Chemosphere 2016;165:144–51. https://doi.org/10.1016/j.chemosphere.2016.09.017.

[91] Meshram P, Dave R, Joshi H, Dharani G, Kirubagaran R, Venugopalan VP. Biofouling control on ultrafiltration membrane through immobilization of polysaccharide-degrading enzyme: optimization of parameters. Desalination Water Treat 2016;57:26861–70. https://doi.org/10.1080/19443994.2016.1168135.

[92] Kim J-H, Choi D-C, Yeon K-M, Kim S-R, Lee C-H. Enzyme-Immobilized Nanofiltration Membrane To Mitigate Biofouling Based on Quorum Quenching. Environ Sci Technol 2011;45:1601–7. https://doi.org/10.1021/es103483j.
